# Optimization of Coagulation-Flocculation with Aluminum Sulfate for the Treatment of Chemical Industry Effluent

**DOI:** 10.1021/acsomega.6c06781

**Published:** 2026-09-28

**Authors:** Sabrina G. Juchem, Suyanne A. L. Bachmann, Fellipe F. S. Farias

**Affiliations:** Federal Institute of Education Science and Technology of Rio Grande do Sul (IFRS), Feliz Campus, Feliz 95770-000, RS, Brazil

## Abstract

Industrial activities require large volumes of water for production processes and for washing equipment, containers, and facilities, generating effluents whose physicochemical characteristics often prevent their direct discharge into receiving water bodies. The treatment and management of industrial wastewater are therefore essential to comply with regulatory standards and minimize environmental impacts. In this context, this study presents a treatability assessment of industrial effluent generated by a chemical products industry employing industrial water treatment located in the Caí Valley region (Rio Grande do Sul, Brazil). The objective was to characterize raw and treated effluents and to propose an efficient treatment strategy based on laboratory-scale evaluations. The methodology included production process mapping, physicochemical characterization of raw effluent, coagulation–flocculation experiments using a Jar-Test under different pH conditions and coagulant dosages, and characterization of the treated effluent under optimized operating conditions. The raw effluent exhibited several parameters above regulatory discharge limits, including pH, phosphorus, turbidity, and iron. Considering regulatory requirements and cost-effectiveness aspects, the optimal treatment conditions consisted of 55 mg/L of 50% aluminum sulfate at an initial pH between 8.0 and 9.0. Under these conditions, significant reductions were observed for several parameters, particularly total alkalinity (85.85%), hardness (85.00%), phosphorus (74.51%), and turbidity (69.03%). Overall, the study demonstrates how specific effluent treatability assessments can support the development of optimized physicochemical treatment configurations, contributing to regulatory compliance and reliable industrial wastewater management.

## Graphical abstract

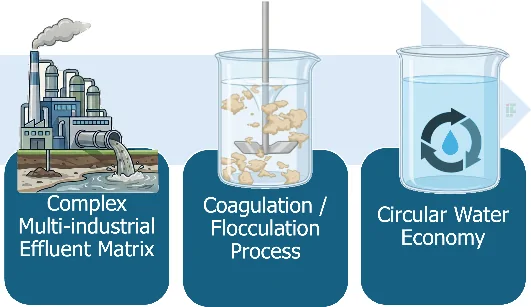

## Introduction

Water resources are of fundamental importance for sustaining life on Earth and are widely used in a broad range of human activities. Among these, agriculture (50.5%), industry (9.4%), and domestic consumption (23.9%) represent the main sectors of water use.^1^ While most of water used in agriculture returns to the atmosphere in the form of vapor, water abstracted for industrial or domestic purposes is discharged into water bodies as effluent, often requiring adequate treatment prior to disposal.^2–5^

Water is also a key resource for economic development, particularly in the industrial sector, where it may be used both as a raw material and as a support medium for production processes. In industrial operations, water is commonly applied as a solvent for solid, liquid, and gaseous substances; for washing and retaining materials in mixtures; and as a heat-transfer fluid in processes involving thermal exchange.^6–8^ In Brazil, the manufacturing sector is responsible for the highest industrial water consumption, especially within the food, beverage, paper and pulp, metallurgy, chemical, and biofuel industries.^9–11^ As industrial activities intensify, both the volume of wastewater generated and its pollution potential increase. According to the Brazilian Water Resources Situation Report, in 2022 the estimated water withdrawal by the manufacturing industry reached 190.52 m^3^ s^–1^, corresponding to 9% of the total water withdrawn nationwide.^10,12^

According to ABNT NBR 9800:1987,^13^ issued by the Brazilian Association of Technical Standards (ABNT), a liquid industrial effluent is defined as ″a liquid discharge originating from an industrial establishment, including industrial process effluents, polluted cooling water, polluted rainwater, and domestic sewage″. The composition and volume of the effluents vary according to the products manufactured, the nature and the capacity of the production process, the degree of modernization and automation, the raw materials employed, and the recycling and reuse practices adopted by the generating source.^14,15^ In general, industrial effluents contain biodegradable organic substances, floating and oily materials, suspended solids, toxic organic compounds, nutrients such as nitrogen and phosphorus, inorganic ions and toxic micropollutants.^16^ These constituents are commonly associated with physicochemical parameters such as color and turbidity (measured in Nephelometric Turbidity Unit – NTU), which may pose risks to the environment and human health if not properly treated. In this context, the analytical characterization of raw and treated effluents is essential for evaluating treatment efficiency and verifying compliance with regulatory standards.^10,13,16–19^

To protect human health and the environment, several laws, resolutions, and decrees regulating effluent discharge into water bodies have been established.^2,3,20^ At the national level, CONAMA (Brazilian National Environment Council) Resolution N° 430, issued on May 13, 2011, defines the conditions and standards for effluent disposal, establishing concentration limits for organic and inorganic parameters that must be met by any generating source. This resolution states that ″effluents may only be discharged, directly or indirectly, into water bodies after proper treatment and in compliance with applicable regulatory requirements″.^21^

Wastewater treatment processes are commonly classified as physical, chemical, and biological, and may be organized into preliminary, primary, secondary, and tertiary stages.^16^ The selection of an appropriate treatment strategy depends on the characteristics of the effluent, the target pollutants, and the quality standards imposed by legislation.^2,17,18,21^ Conventional wastewater treatment widely relies on biological processes, which typically employ aeration to supply oxygen to microorganisms that degrade organic matter. Aeration is also conventionally applied as a physical step to promote the oxidation and subsequent precipitation of dissolved metals, such as iron and manganese. Alternatively, conventional physicochemical treatment consists of a series of steps, including coagulation, flocculation, sedimentation, and rapid or slow filtration. While biological treatments are highly efficient, physicochemical approaches, such as coagulation-flocculation, are widely favored as a primary treatment or standalone strategy due to their operational simplicity, cost-effectiveness, and rapid response.^22–25^ In practice, efficient treatment systems often rely on a combination of different processes. In this study, the evaluation focused on the physicochemical process, as it provides a robust and highly feasible alternative for bench-scale treatability assessments, allowing for a precise optimization of the removal of suspended solids, colloidal particles, and associated contaminants prior to discharge.

Among chemical treatment methods, coagulation/flocculation plays a central role in the removal of suspended solids, colloidal particles, and associated contaminants. This process involves the addition of coagulants and pH-adjusting agents to promote the formation of hydrolyzed cationic species, which destabilize negatively charged particles and facilitate their aggregation into larger flocs. Subsequent sedimentation or flotation enables the separation of these flocs from the treated effluent.^22–24,26–28^ Coagulation-flocculation is a widely used technology in the treatment of industrial effluents, where the main mechanism is due to the neutralization of the charges of negatively charged colloids by hydrolyzed cationic products, which allows the aggregation of particles through van der Waals forces, forming micro flocs. The efficiency of the process depends on several factors such as the type of coagulant, dosage, pH, temperature, ionic strength, nature and concentration of organic matter, total dissolved solids, and the distribution of suspended colloidal particles.^22,26,28,29^

Among coagulants, aluminum sulfate is the most widely used in the physicochemical treatment of industrial effluents due to its simplicity and cost-effectiveness, being efficient in removing suspended particles, organic matter, heavy metals, and other impurities. Determining the dosage of aluminum sulfate is fundamental in the design and operation of an effluent treatment system, being considered a critical parameter in determining treatment efficiency.^30,31^ Because the composition of industrial effluents varies widely depending on the production process, the selection and optimization of treatment technologies require specific treatability assessments. Although coagulation–flocculation is widely applied in industrial wastewater treatment, the efficiency of this process strongly depends on the physicochemical characteristics of each effluent, making laboratory-scale evaluations essential prior to full-scale implementation.

Previous studies have demonstrated the efficacy of this approach. Haydar and Aziz conducted a study aimed at treating tannery effluent through coagulation, flocculation, and sedimentation with aluminum sulfate. Using the jar-test, they achieved a removal of 97% of turbidity, 93.5% of total suspended solids (TSS), 36.2% of chemical oxygen demand (COD), and 98.4% of chromium.^32^ Dotto et al. used coagulation/flocculation for the treatment of textile effluent, comparing the efficiency of a natural coagulant with aluminum sulfate. In this study, even though organic coagulant obtained greater efficiency than the inorganic coagulant, aluminum sulfate showed a turbidity removal of 87.6% and COD removal of 73.6%.^33^ Couto Junior, Barros and Pereira carried out physicochemical treatment tests on textile effluent with tannin and aluminum sulfate, in which the latter showed 93.12%, 99.06%, and 99.29% removal of COD, color and turbidity, respectively.^34^

Currently, the specific effluent investigated in this study is managed through an internal recycling system that allows for its reuse in specific industrial activities. However, because this chemical industry specializes in providing water and effluent treatment products and services for various other industrial sectors, the effluent generated at their facility, primarily from washing multipurpose equipment and chemical containers, is a highly complex and variable matrix. It contains a wide mixture of contaminants from diverse industrial origins. As the company continues to expand, the volume of generated effluent will inevitably increase, necessitating its direct discharge into receiving water bodies in the near future. This creates a significant environmental and operational challenge: identifying a versatile, technically feasible, and cost-effective treatment alternative capable of handling this complex matrix to meet strict regulatory discharge standards.

Among the available alternatives, conventional coagulation-flocculation using aluminum sulfate was selected due to its recognized efficiency, operational simplicity, and low cost. While this method is widely used, its efficacy on nonstandard, highly variable wastewater profiles requires specific treatability assessments. In this context, this study presents a comprehensive treatability assessment of this complex, multiorigin effluent generated by a chemical products industry in the Caí Valley region (Rio Grande do Sul, Brazil).

The novelty and distinctiveness of this work lie in demonstrating how a conventional, low-cost physicochemical approach can be effectively optimized, by determining the optimal pH and coagulant dosage, to successfully treat an extremely complex industrial matrix. In addition to providing a robust technological solution for the company’s future environmental compliance, this study establishes a practical and reliable framework for treating complex effluents generated by environmental service providers.

## Materials and Methods

### Company Characterization

The company commercializes chemical products for the treatment of water, influents, effluents, and industrial sanitizers, serving clients throughout southern Brazil. Its main clients operate in the food industries (e.g., dairies, meat processing plants, confectionery, and pickling), textiles, furniture, plastics, footwear, tanneries, lumber mills, metallurgy, and the healthcare sector. The services provided by the company include technical assistance in industrial water treatment, operational optimization tests for equipment, water quality monitoring through physicochemical analyses and the issuance of technical reports with recommendations for process improvement.

### Materials

This section describes the materials and equipment used for effluent characterization and Jar-Test assays. The following parameters were evaluated: total alkalinity, hardness, chlorides, chemical oxygen demand (COD), iron, phosphorus, sodium, potassium, pH, electrical conductivity, total dissolved solids (TDS), settleable solids, dissolved oxygen (DO), and turbidity.

For the determination of total alkalinity, a standardized sulfuric acid solution (H_2_SO_4_, Top Glass, 0.05 N) was used, alongside with a mixed indicator composed of bromocresol green and methyl red (Top Glass). Hardness was determined using a standard ethylenediaminetetraacetic acid solution (EDTA, Merse, 0.01 M), a 15% ammonium buffer solution (Top Glass), and Eriochrome Black T indicator (Neon). For chloride determination, silver nitrate (AgNO_3_, Top Glass, 0.1 M) was used as a standard solution, with potassium chromate (K_2_CrO_4_, Top Glass, 5% w/v) as the end point indicator. COD was determined using potassium permanganate (KMnO_4_, Cinética, 0.002 M) as the standard oxidizing solution and sodium oxalate (Na_2_C_2_O_4_, Synth, 0.005 M) as the equivalence indicator, along with a 25% v/v sulfuric acid solution (Neon).

For the digestion and subsequent neutralization of iron and phosphorus, a commercial reagent kit was employed (Nanocolor Nanox Metal Kit, reference 918978, Macherey-Nagel). Phosphorus analysis was carried out using reagents from the Nanocolor Ortho-Phosphate Kit (reference 91878, Macherey-Nagel), while iron concentration was determined using the reagents from the Visocolor Powder Pillows Kit (reference 936227, Macherey-Nagel).

Sodium and potassium concentrations were determined via flame spectrophotometry. For this purpose, stock solutions at a concentration of 1000 mg L^–1^ of sodium, potassium, and lithium were prepared using analytical-grade reagents: sodium chloride (Química Moderna, 99.5% purity), potassium chloride (Química Moderna, 99% purity), and lithium carbonate (Dinâmica, 99% purity).

For the Jar-Test assays, a 25% sulfuric acid solution (Neon) was used for pH adjustment, and aluminum sulfate (50%, MK Química) was applied as the coagulant. The equipment used for physicochemical analyses and experiments included: a Digimed pH meter (model DM-22); an AZ Instrument Corp. conductivity meter (model 86505 AZ EB); a SolidSteel thermostatic bath (model SSD); a Macherey-Nagel UV–vis spectrophotometer (model 919650.1); a Macherey-Nagel digestion block (model 919380); a LineLab oximeter (Do Eco model); a Del Lab turbidimeter (model DLT-JE); a Milan Jar-Test apparatus (model 303–6); an Imhoff cone (Sigma-Aldrich); and a flame spectrophotometer prototype developed by Institute’s faculty as part of a research project.

### Sampling

For sampling, 15 L of effluent were collected in previously sanitized polyethylene terephthalate containers (washed with neutral detergent, tap water, and rinsed with distilled water). A simple grab sampling method was used, with the effluent collected at a single point in time from the storage cistern. The collect effluent was kept refrigerated to preserve its initial physicochemical characteristics.

### Physicochemical Characterization

The physicochemical characterization of the raw and treated effluent was performed using classical and instrumental analytical techniques, following standard analytical procedures. Total alkalinity, hardness, chlorides, and COD were determined by volumetric titration methods. Parameters such as pH, electrical conductivity, TDS, iron, phosphorus, sodium, potassium, DO, and turbidity were measured using instrumental techniques. The analyses were conducted in the company’s laboratory, as well as in the Chemical Engineering and Chemistry and Environment Laboratories at the Federal Institute of Rio Grande do Sul (IFRS) – Feliz Campus. All measurements were performed in triplicate to ensure analytical reliability.

Table 1 summarizes the titrimetric analytical methods employed for the determination of physicochemical parameters of the raw and treated effluents. The methods were based on Standard Methods for the Examination of Water and Wastewater,^35^ issued by the American Public Health Association (APHA), with minor adaptations made to accommodate laboratory-scale conditions, sample volume constrains, and reagent availability, without compromising accuracy.

**Table 1:** Titrimetric Methods Used for the Determination of Physicochemical Parameters of the Effluent under Study

Parameter	Method (titrimetric)	Adaptations
Total Alkalinity (ppm of CaCO_3_)	2320 B	Used 0.05 N H_2_SO_4_ standard solution and a mixed indicator.
Hardness (ppm of CaCO_3_)	2340 C	Sample not pretreated with acid; 50 mL sample volume; no inhibitor used to improve end point visualization.
Chlorides (ppm of Cl^–^)	4500-Cl^–^ B	50 mL sample volume; used 0.1 M AgNO_3_ standard solution.
COD (mg/L of O_2_)	5220 C	Used KMnO_4_ as both the oxidizing agent and standard solution; digestion at 75 °C for 30 min in a thermostatic bath; Na_2_C_2_O_4_ used as end point indicator.

Reference: Adapted from APHA (2012).^35^

In addition to titrimetric analyses, instrumental analytical techniques were employed to determine parameters that require higher sensitivity, selectivity, or rapid response. Table 2 presents the instrumental methods used, their respective references, and the adaptations applied,

**Table 2:** Instrumental Analytical Methods Used for the Determination of Physicochemical Parameters of the Effluent under Study

Parameter	Method	Adaptation
pH^a^	4500-H^+^ B (Potentiometric)	-
Conductivity(μS/cm or ms/cm)^a^	2110 B (Conductometric)	-
Total dissolved solids (mg/L)^a^	2510 A (Conductometric)	-
Settleable solids (mg/L)^b^	NBR 10561 (Gravimetric)	500 mL sample volume used.
Iron (ppm)^a^	3030 D(Sample digestion)3500-Fe B (Colorimetric)	6 mL sample digested at 120 °C in a block; 2.5 mL digested sample analyzed using the iron kit reagent.
Phosphorus (ppm)^a^	3030 D(Sample digestion)4500-PC (Colorimetric)	8 mL sample digested at 120 °C in a block; 2 mL digested sample analyzed with 0.4 mL of R1 and R2 kit reagents.
Dissolved Oxygen(mg/L)^a^	4500-OG (Electrochemical)	-
Turbidity (NTU)^c^	Nephelometric	-

a Used or Adapted from APHA (2012).^35^

b Adapted from Brazilian Association of Technical Standards (ABNT - 1988).^13^

c Used from National Health Foundation (FUNASA – 2013).^36^

The combination of classical titrimetric methods and instrumental techniques allowed for a comprehensive analytical assessment of the effluent, ensuring data accuracy, reproducibility, and compliance with regulatory standards.

### Analysis of Sodium and Potassium by Flame Spectrophotometer

For the determination of sodium and potassium, a flame atomic emission spectrophotometer was employed, using the standard dilution method described by Jones et al. (2015).^37^ Stock reference solutions with a concentration of 1000 mg/L were prepared by dissolving analytical-grade metal salts (sodium, potassium, and lithium) in deionized water obtained from a reverse osmosis system coupled to a deionizer.

For sodium determination, two solutions were prepared for each sample. Solution A contained 500 μL of the sample supplemented with sodium, potassium, and lithium, while solution B contained 500 μL of the sample only. Both solutions were diluted to a final volume of 50 mL with deionized water. For potassium determination, the same procedure was followed; however, sodium was not added to Solution A.

Flame spectrophotometric measurements were performed using the same procedure for both sodium and potassium. Initially, the contents of Solution A were transferred to a larger container and aspirated into the instrument. After the signal reached a stable value for all elements, the contents of Solution B were added to the same container, and the change in signal intensity during dilution was continuously recorded. Lithium, which is typical absent from environmental samples, was used as an internal standard.

To determine the analyte concentrations, a calibration plot (Supporting Information - SI) was constructed by plotting the ratio of the analyte signal to the internal standard signal (*y*-axis) as a function of the inverse of the internal standard concentration (*x*-axis). The concentration of sodium or potassium in the sample was calculated from the ratio between the slope and the y-intercept of the resulting linear relationship.

### Jar-Test Assays

Coagulation/flocculation and sedimentation assays were performed using a Jar-Test apparatus, a laboratory-scale benchtop batch unit. Figure 1 shows the Milan Jar-Test, model 303–6, employed in this study. This equipment consists of six jars with a capacity of 2.0 L each, equipped with rotating paddles that allow precise control of rotation speed.

**Figure 1:**
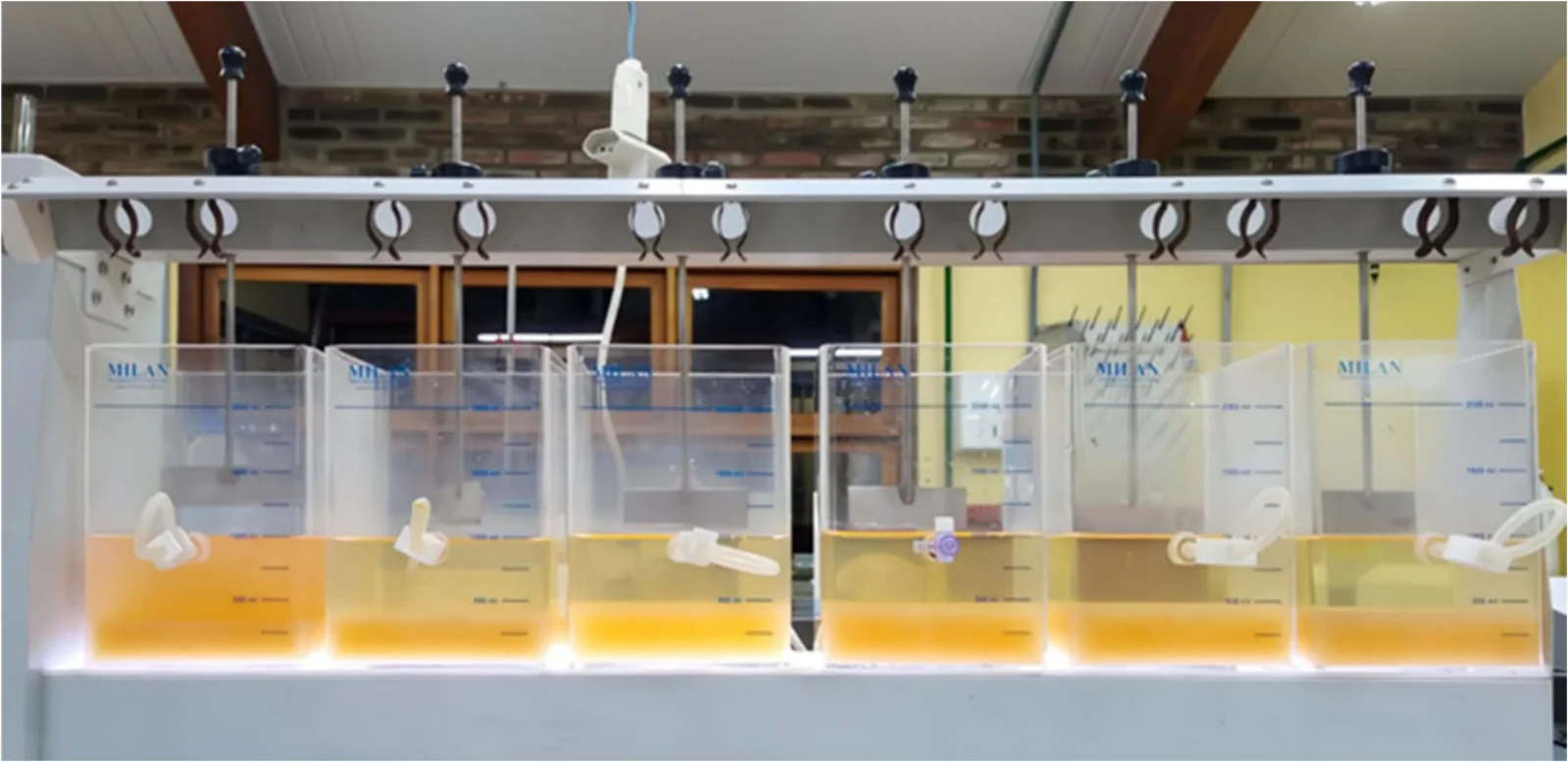
Milan Jar-Test, model 303–6.

The experimental procedure was based on the methodology proposed by the National Health Foundation from Brazil.^36^ Initially, each jar was filled with 1000 mL of raw effluent, and the pH was adjusted using with a 25% sulfuric acid solution. The Jar-Test was then operated at a rapid mixing speed of 100 rpm, at which point the calculated dosages of the coagulant (50% aluminum sulfate) were added to each jar using a micropipette. Rapid mixing was maintained for 2 min and 30 s to ensure proper dispersion of the coagulant.

Subsequently, the agitation speed was reduced to 50 rpm and maintained for 25 min to promote flocculation. After this period, agitation was stopped, and the samples were allowed to settle for an additional 25 min. At the end of the sedimentation step, the supernatant from each jar was carefully collected, and pH, turbidity, and total alkalinity were determined in triplicate to identify the optimal coagulant dosage.

Two sets of Jar-Test assays were conducted. In the first set, six jars were used with the initial effluent pH adjusted between 6 to 7, and aluminum sulfate dosages of 25, 30, 35, 40, 45, and 50 mg/L were evaluated. The second set was performed using four jars, with the initial pH adjusted between 8 to 9 and coagulant dosages of 45, 50, 55, and 60 mg/L.

It is worth noting that the pH range between 7 and 8 is considered optimal for the occurrence of the sweep coagulation mechanism, according to the aluminum sulfate coagulation diagram (Figure S1 - Supporting Information).^38^

### Statistical Analysis

The experimental results obtained for the efficiency indicators of the Jar-Test assays (pH, turbidity, and total alkalinity) were statistically evaluated using a one-way analysis of variance (ANOVA) considering a 95% confidence level (α = 0.05). Statistical analyses were performed using Microsoft Office Excel 365 software.

### Evaluation of Physicochemical Treatment Efficiency

The efficiency of the physicochemical treatment was assessed by comparing the characterization parameters of the raw effluent with those obtained after treatment. Treatment efficiency was calculated using eq (1).

1 $$E=\frac{C_{o}-C_{E}}{C_{o}}\times 100$$

where:

*E* = the treatment efficiency (%)
*C*_0_ = the initial concentration of the parameter, expressed in its respective unit
*C*_E_ = the final concentration of the parameter after treatment, expressed in its respective unit

## Results and Discussion

The methodology adopted in this study follows a case study approach and was structured into four main stages, as illustrated in Figure 2.

**Figure 2:**
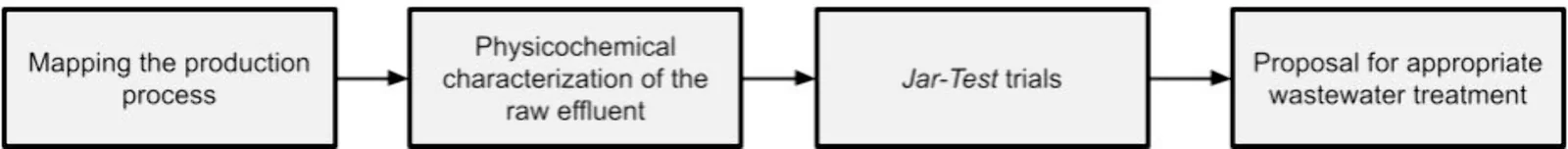
Stages of the study development.

The first stage focused on mapping the industrial production process, in order to identify the operational steps responsible for effluent generation. Given that the company manufactures a wide range of chemical products, a detailed survey was conducted to identify the main products used in industrial water treatment, which are directly associated with the characteristics of the generated effluents.^10,29^

This mapping step was essential to understand the origin, variability, and potential contamination sources of the effluent, thereby providing a technical basis for the selection of analytical parameters and for the subsequent treatability studies. Moreover, identifying the stages responsible for effluent generation allows for a more targeted evaluation of treatment strategies and facilitates the extrapolation of the proposed approach to other industries with similar operational profiles.^16,29^

### Production Process and Effluent Generation

The company is organized into four main sectors: administrative, technical, laboratory, and production. The administrative and technical sectors generate effluents with characteristics similar to domestic wastewater, which are directed to the septic tank and, therefore, are not included in the scope of this study.

In the laboratory sector, physicochemical analyses of industrial water are routinely performed, including measurements of pH, alkalinity, hardness, among others. During these analyses, aqueous samples containing chemical reagents are discarded through sinks connected to a sewage system that leads to a storage cistern, where the effluent is retained until treatment. Although the volume of effluent generated in this sector is relatively low, its chemical composition may vary depending on the reagents used in the analyses.

The production sector represents the main source of industrial effluent generation. The production process comprises four main stages: (i) addition of raw materials to agitation tanks, (ii) agitation and mixing, (iii) bottling, and (iv) shipping. In addition, containers returned by customers containing residual chemical products are washed for reuse, and the resulting wash water is directed to the same storage cistern. Floor washing in the production area also contributes to the total effluent volume. Figure 3 presents a flowchart of the company’s activities, highlighting the stages associated with industrial effluent generation.

**Figure 3:**
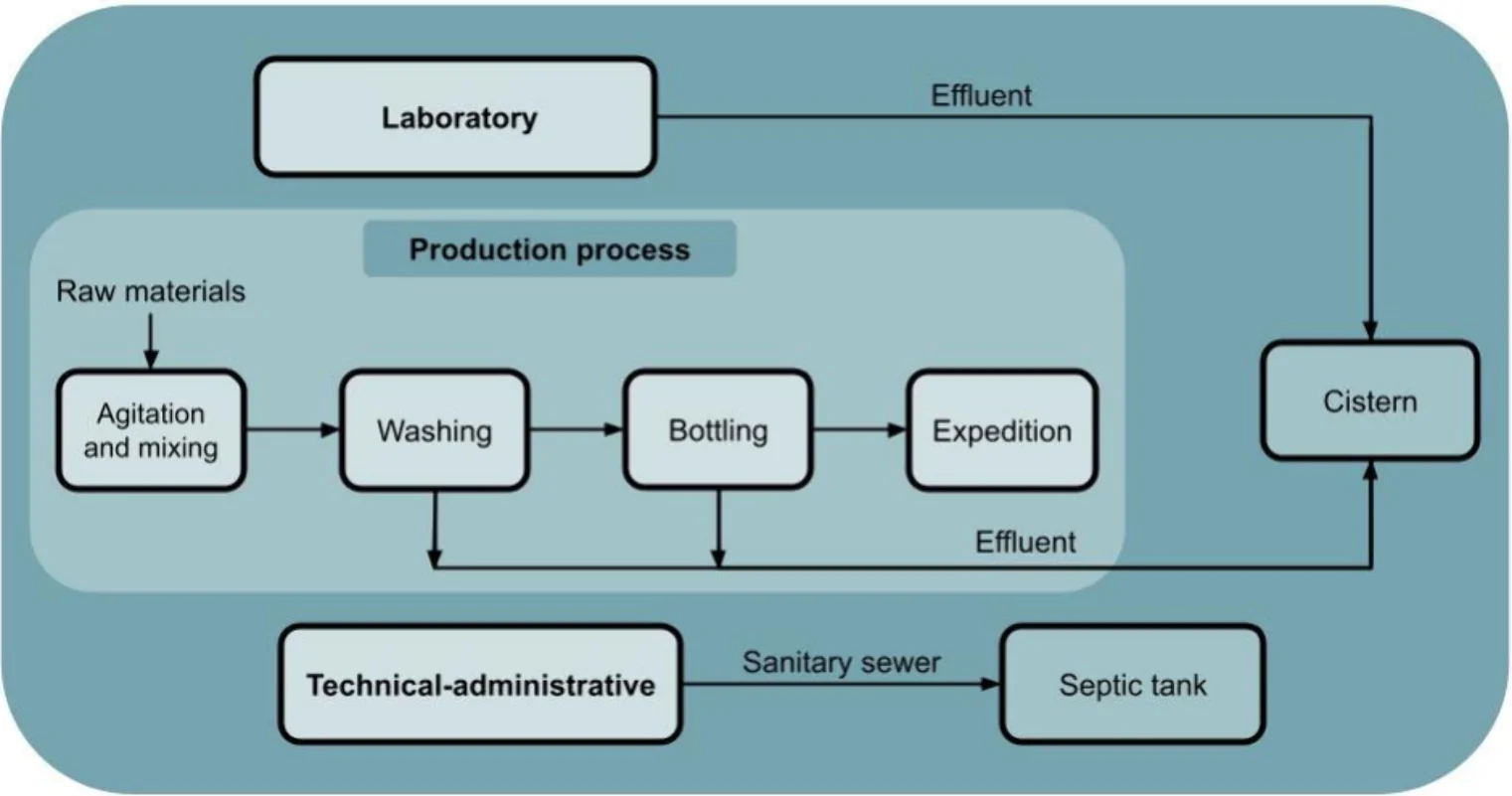
Flowchart indicating the main stages of the production process and the contributing factors to industrial effluent generation.

It is important to emphasize that the objective of this study is limited to the industrial effluent, which comprises wastewater generated in the laboratory and production sectors. The characteristics of this effluent are strongly influenced by the formulations of the chemical products manufactured by the company.

Table 3 summarizes the main products produced and their respective raw materials. These products can be grouped into five categories: organic, alkaline, metal-based, antifoaming agents, or others. Organic products are mainly composed of tannins. Alkaline formulations contain sodium hydroxide as the primary raw material, typically in large quantities. Metal-based products include ferric chloride and aluminum sulfate. Antifoaming agents are predominantly composed of silicone and fatty acids. Additionally, other formulations are produced using diverse raw materials, such as acids, amines, and oxides, generally in smaller proportions.

**Table 3:** Main Products Manufactured by the Company and Their Respective Raw Materials

Classification	Raw materials	Formulations
Organic	Tannin + formaldehyde + water	Product A
Alkalis	Sodium tripolyphosphate + sodium sulfite + sodium polyacrylate + sodium hydroxide + amine blend + water	Product B
	Sodium tripolyphosphate + sodium sulfite + sodium polyacrylate + sodium hydroxide + water	Product C
	Polymaleic acid + sodium hydroxide + water	Product D
	Sodium polyacrylate + sodium hydroxide + water	Product E
	Sodium hydroxide + water	Product F
	Carboxylic polymers + sodium sulfite + sodium hydroxide + amine blend + water	Product G
	Sodium hypochlorite + water	Product H
Metals	Aluminum polychloride + polyacrylamide + water	Product I
	Aluminum polychloride + water	Product J
	Aluminum sulfate + water	Product K
	Aluminum sulfate + ferric chloride + water	Product L
	Ferric chloride + water	Product M
Antifoaming agents	Silicone + fatty alcohols + water	Product N
Others	Trimethylphosphonic acid + sodium polyacrylate + zinc oxide + water	Product O
	Amine blend + water	Product P
	Technical grade urea + water	Product Q
	Hydrochloric acid + water	Product R

Despite the wide variety of chemical products manufactured by the company (Table 3), most formulations are predominantly inorganic in nature, even though they are applied in the physicochemical treatment of industrial water. Only a limited number of products contain organic components, mainly tannins or organic polymers.

Given this compositional profile, physicochemical treatment is preferentially recommended over biological treatment.^16^ Physicochemical processes are particularly effective for the removal of inorganic pollutants, heavy metals, oils and greases, color, settleable solids, and suspended solids, whereas biological treatment is primarily aimed at the removal of carbonaceous organic matter and nutrients. Nevertheless, detailed effluent characterization remains essential to support and validate this treatment strategy.

Moreover, for biological treatment to be technically viable, adequate conditions of pH, biodegradable organic matter, and nutrients - especially, nitrogen, phosphorus, and sulfur – must be ensured to sustain microbial activity. Insufficient nutrient availability significantly reduces the efficiency of biological processes. While domestic wastewater typically presents nutrient supplementation to achieve effective biodegradation.

COD is commonly used as an indicator of organic matter content. Low COD values indicate substrate limitation for microbial growth and maintenance. In domestic sewage, COD values typically range from 250 mg/L for weak sewage to 1000 mg/L for strong sewage. Therefore, effluents presenting COD values below this range may be considered excessively diluted, rendering biological treatment inefficient.

As shown in Table 3, only a few of chemical products contain nutrients relevant to biological treatment. Furthermore, the characterization of the raw effluent, presented in a subsequent section, revealed a low COD value (232.00 ± 8.00 mg/L). This result confirms that biological treatment would likely be inefficient for the effluent under study, thereby reinforcing the selection of physicochemical treatment as the most appropriate approach.

### Determination of pH and Coagulant Dosage

Jar tests were conducted to determine the optimal pH range and aluminum sulfate dosage for the physicochemical treatment of the studied effluent. The results obtained for initial and final pH, turbidity, and total alkalinity are presented in Table 4. Jars 1 to 6 were processed in a single batch, while jars 7 to 10 were evaluated in a second batch due to the equipment capacity limitation.

**Table 4:** Jar-Test Results for the Evaluation of Aluminum Sulfate Dosage and pH Conditions, Including Initial and Final pH, Turbidity, and Total Alkalinity of the Treated Effluent

Entry	Dosage, mg/L	Initial pH	Final pH	Final turbidity (NTU)	Final Total Alkalinity (ppm of CaCO_3_)
1	25	6.99	6.42 ± 0.03	117.00 ± 3.00	230.00 ± 8.66
2	30	6.40	6.00 ± 0.02	17.20 ± 0.91	146.67 ± 5.77
3	35	6.58	6.04 ± 0.04	8.84 ± 1.09	151.67 ± 5.77
4	40	6.91	6.16 ± 0.02	11.40 ± 0.57	166.67 ± 14.43
5	45	6.71	5.92 ± 0.10	5.73 ± 0.42	130.00 ± 5.00
6	50	6.45	5.59 ± 0.02	4.59 ± 1.21	90.00 ± 5.00
7	45	8.12	6.68 ± 0.15	107.67 ± 1.53	256.67 ± 5.77
8	50	8.72	7.50 ± 0.02	130.67 ± 1.15	335.00 ± 5.00
9	55	8.66	7.05 ± 0.02	26.53 ± 3.45	268.33 ± 7.64
10	60	8.76	7.32 ± 0.09	48.77 ± 1.24	295.00 ± 10.00

Prior to coagulation/flocculation, pH adjustment was required to ensure more efficient treatment. In the first batch (jars 1–6), the adjusted initial pH values were lower than the target range of 8–9. The subsequent addition of aluminum sulfate further reduced the pH, resulting in final values below the limits established by state legislation (pH 6–9), according to State Council for the Environmental (CONSEMA – Rio Grande do Sul) Resolution 355/2017.^39^ This condition would be unfavorable for potential effluent discharge, as it would require additional pH correction and increased chemical consumption.

Among the first batch, jar 6 (50 mg/L) presented the lowest turbidity and total alkalinity values, a result that was also visually evident, as shown in Figure 4, when compared to the raw effluent.

**Figure 4:**
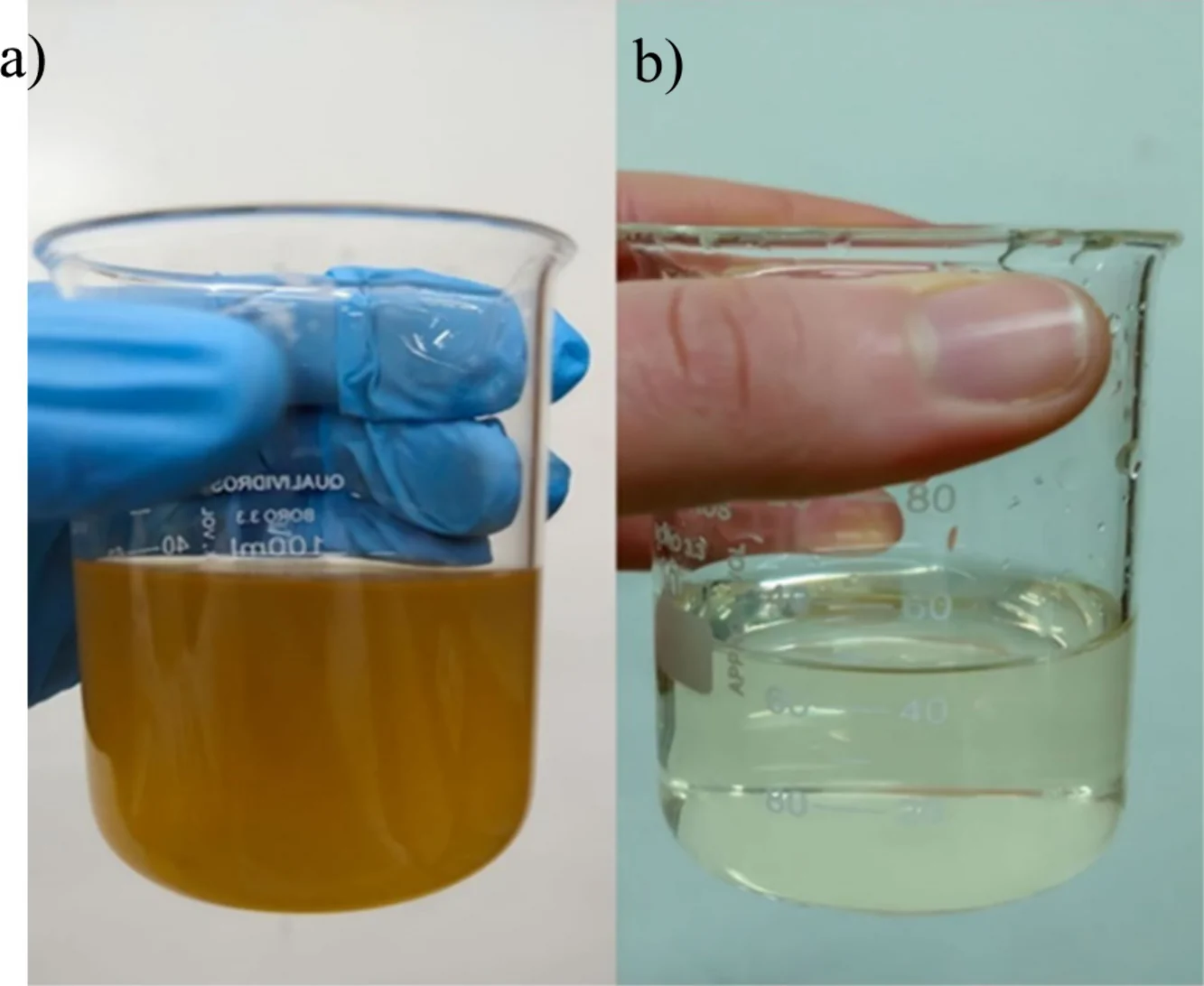
Picture of the a) raw effluent and b) the treated effluent under the operating conditions of jar 6.

In the second batch (jars 7–10), jar 9 (55 mg/L) exhibited the most satisfactory performance, achieving the lowest turbidity and total alkalinity values while maintaining a final pH within the limits established by both state and federal regulations.

To statistically evaluate the treatment performance, one-way ANOVA tests were applied (SI). For jars 6 and 9, corresponding to aluminum sulfate dosages of 50 and 55 mg/L, respectively, the analysis indicated statistically significant differences between group means. The calculated F values for pH (F = 9504.8), total alkalinity (F = 1144.9), and turbidity (F = 107.80) were higher than the critical F value (7.7086), demonstrating greater variability between groups than within groups. In addition, the p-values for pH (6.63 × 10^–8^), turbidity (4.86 × 10^–4^), and total alkalinity (4.55 × 10^–6^) were all below the significance level (α = 0.05), confirming significant differences among treatments.

However, the selection of the optimal coagulant dosage must also consider compliance with environmental legislation and treatment costs. Table 5 summarizes the regulatory limits established by state and federal standards. It should be noted that no legal limit is defined for total alkalinity.

**Table 5:** Parameters of Federal and State Legislation for Efficiency Indicators

Parameter	CONAMA Resolution 430/2011	CONAMA Resolution 357/2005	CONSEMA Resolution 355/2017
pH	5 – 9	6 – 9	6 – 9
Turbidity	-	100 NTU	-

Although a dosage of 50 mg/L (jar 6) resulted in lower turbidity and alkalinity values, the final pH fell below the limits established by state legislation. Additionally, a higher volume of sulfuric acid was required for pH adjustment, increasing operational costs.

Therefore, considering regulatory compliance and cost-effectiveness, the most suitable treatment condition was defined as 55 mg/L of aluminum sulfate, with an initial pH range between 8 and 9. Under these conditions, the final pH complies with both state and federal legislation, and turbidity values remain within the limits established by CONAMA Resolution 357/2005.^40^ Furthermore, the lower consumption of sulfuric acid results in a more favorable cost–benefit ratio for the company. Although the coagulant dosage is slightly higher, the cost of aluminum sulfate (1.28 kg^–1^ in Brazilian reais or 0.25 kg^–1^ in US dollars, at actual currency) is significantly lower than that of sulfuric acid (4.35 in Brazilian reais -0.83 in US dollars).

Based on these results, an additional jar-test assay was performed to evaluate the reproducibility of the physicochemical treatment under the selected optimal conditions (55 mg/L of aluminum sulfate and initial pH between 8 and 9).

### Efficiency of the Physicochemical Treatment

Table 6 presents the characterization results of the raw and treated effluent obtained under the optimal operating conditions. A significant improvement in effluent quality was observed after physicochemical treatment. Dissolved oxygen increased from 4.07 ± 0.15 mg/L in the raw effluent to 7.23 ± 0.29 mg/L after treatment, corresponding to an increase of 77.87%. Although CONAMA Resolution 430/2011 does not establish limits for this parameter, CONAMA Resolution 357/2005 sets a minimum dissolved oxygen concentration of 5 mg/L for Class II freshwater, which includes waters intended for human consumption after conventional treatment.^40^ In accordance with United States (US) and European Union (EU) legislation, dissolved oxygen must also be greater than or equal to 5 mg/L for the disposal of industrial effluents.^41,42^ The observed increase in dissolved oxygen can be mainly attributed to the agitation stages of the physicochemical treatment, which enhance oxygen transfer to the liquid phase. This improvement is environmentally relevant, as dissolved oxygen is essential for the maintenance of aquatic life.^43^

**Table 6:** Results of the Characterization of Raw and Treated Effluent, Legal Parameters and Efficiency of the Physical-Chemical Treatment

Parameter	Raw effluent	Treated effluent^a^	CONAMA 430/2011	CONAMA 357/2005	CONSEMA 355/2017	Efficiency, %
Total Alkalinity^c^	1833.33 ± 11.55	259.44 ± 12.62	-	-	-	85.85
Hardness^c^	400.00 ± 0.01	60.00 ± 0.01	-	-	-	85.00
Chlorides^c^	1514.67 ± 20.50	1420.00 ± 35.50	-	250	-	6.25
COD^d^	232.00 ± 8.00	112.00 ± 8.00	-	-	330	51.72
pH	11.60 ± 0.06	6.94 ± 0.15	5 – 9	6–9	6–9	-
Turbidity^e^	117.67 ± 1.15	36.44 ± 12.29	-	100	-	69.03
Dissolved Oxygen^d^	4.07 ± 0.15	7.23 ± 0.29	-	>5	-	77.87
Conductivity^f^	7996.67 ± 96.09	6473.33 ± 45.09	-	-	-	19.05
Total dissolved solids (TDS)^d^	6557.27 ± 78.79	5308.13 ± 36.98	-	500	-	19.05
Settleable solids (SS)^g^	0.01 ± 0.01	0.01 ± 0.01	<1	-	≤1	^b^
Sodium^d^	2045.00 ± 0.01	1819.33 ± 31.94	-	-	-	11.04
Phosphorus^c^	17.39 ± 0.75	4.43 ± 0.77	-	0.030–0.050	4	74.51
Iron^c^	>6.00 ± 0.01	2.71 ± 1.59	-	-	10	>54.89
Potassium^d^	7.2 ± 0.01	5.27 ± 1.03	-	-	-	26.81

a Units: ppm.

b mg/L of O_2_.

c NTU.

d μS/cm.

e mL/L.

f Aluminum sulfate dosage of 55 mg/L and pH of 6.67 ± 0.24 to 8.57 ± 0.30.

g The calculation could not be completed due to the method’s detection limit.

There are also international laws that establish limits for the discharge of industrial effluents. Table 7 presents the current legislation in the US and EU and their respective parameters.

**Table 7:** US and EU Parameters for the Disposal of Industrial Effluents

Parameter	US limits^a^	EU limits
Chlorides	230 mg/L	250 mg/L(Directive 98/83/EC)
COD	250 – 1000 mg/L	≤125 mg/L(Directive 91/271/EEC)
pH	6 – 9	6 – 9(Directive 91/271/EEC)
Dissolved oxygen	≥5 mg/L	≥5.0 mg/L(Directive 2000/60/EC)
Conductivity	-	250 – 1000 μS/cm(Directive 2000/60/EC)
Total dissolved solids	250 – 2.500 mg/L	-
Phosphorus	0.1 – 1.0 mg/L	1.0 – 2.0 mg/L(Directive 91/271/EEC)
Iron	<1.0 mg/L	-

a EPA water quality criteria.^42^

The pH showed a reduction of 4,66 units, decreasing from highly alkaline values in the raw effluent (11.60 ± 0.06) to near-neutral values after treatment (6.94 ± 0.15). This final pH remains within the limits established by both federal and state legislation (pH 6.0 – 9.0), which is also consistent with the discharge limits set by the United States and the European Union.^41,42^ pH adjustment was required prior to the jar-test assays because aluminum sulfate exhibits higher efficiency via the sweep coagulation mechanism in the pH range between 7.0 and 8.0 (Figure S1 - Supporting Information). Furthermore, the pH adjustment is required for efficient physicochemical treatment via coagulation/flocculation to guarantee the hydrolyzed aluminum sulfate species formation. These species are necessary to destabilize the pollutant charges, enabling their agglomeration and the formation of flocs so that they can, subsequently, be separated via the sedimentation process. In physicochemical treatment processes, complex chemical reactions occur, making it essential to determine both the optimal pH and the appropriate coagulant dosage to promote effective separation of suspended and colloidal pollutants. Furthermore, pH directly influences aluminum speciation and its precipitation as hydroxides; when pH conditions are inadequate, aluminum species may remain solubilized, impairing the overall coagulation efficiency. Therefore, the final optimal pH achieved (6.94) is highly favorable for the precipitation of aluminum as hydroxides, which minimizes the concentration of residual soluble aluminum in the treated effluent.

A marked reduction was also observed for other parameters, especially total alkalinity (85.85%), hardness (85.00%), phosphorus (74.51%), and turbidity (69.03%). Total alkalinity is directly related to pH buffering capacity and the presence of carbonate and bicarbonate species. Its decrease is expected during treatment with aluminum-based coagulants, since aluminum sulfate consumes alkalinity during hydrolysis and floc formation. During this process, two distinct mechanisms act synergistically to remove impurities. First, the dissociated Al^3+^ ions and their subsequent soluble cationic species act as the primary coagulating agents, neutralizing the negative surface charges of the colloidal particles. Simultaneously, the reaction between aluminum sulfate and bicarbonate alkalinity produces aluminum hydroxide precipitates (Al­(OH)_3_). These solid precipitates act via the sweep-floc mechanism, enmeshing the destabilized particles into denser flocs that rapidly settle out of suspension. The overall precipitation reaction is represented by Equation 1a:

1a $${\mathrm{Al}}_{2}{\left({\mathrm{SO}}_{4}\right)}_{3}\cdot 18H_{2}O+3\mathrm{Ca}{\left({\mathrm{HCO}}_{3}\right)}_{2}\to 2\mathrm{Al}{\left(\mathrm{OH}\right)}_{3}+3{\mathrm{CaSO}}_{4}+6{\mathrm{CO}}_{2}+18H_{2}O$$

Total alkalinity is also related to water hardness, since the most common cations associated with alkalinity-forming anions are Ca^2+^ and Mg^2+^, which are also responsible for hardness. According to hardness classification, water can be considered soft (<50 mg/L CaCO_3_), moderately hard (50–150 mg/L CaCO_3_), hard (150–300 mg/L CaCO_3_), or very hard (>300 mg/L CaCO_3_).^44^ As shown in Table 6, the raw effluent presented very high alkalinity (1833.33 ± 11.55 mg/L CaCO_3_) and hardness (400.00 ± 0.01 mg/L CaCO_3_), being classified as very hard water. After physicochemical treatment, alkalinity was reduced to 259.44 ± 12.62 mg/L CaCO_3_ and hardness to 60.00 ± 0.01 mg/L CaCO_3_, corresponding to removal efficiencies of 85.85% and 85.00%, respectively, resulting in moderately hard water. Although neither alkalinity nor hardness have limits established by current legislation, their substantial reduction is environmentally and operationally relevant.

Regarding COD, the raw effluent presented a concentration of 232.00 ± 8.00 mg/L O_2_, which is below the maximum limit of 330 mg/L established by CONSEMA Resolution 355/2017 for effluent discharge flows below 100 m^3^/day. For COD, the United States sets a maximum limit of 250 to 1000 mg/L, while the European Union allows a value lower than 125 mg/L. After treatment, COD was reduced to 112.00 ± 8.00 mg/L O_2_, corresponding to a removal efficiency of 51.72%. COD is commonly used as an indicator of organic matter content, representing the amount of oxygen required for the chemical oxidation of organic compounds. Although organic matter is essential for aquatic ecosystems, excessive concentrations may cause undesirable effects such as increased color, odor, turbidity, and depletion of dissolved oxygen due to microbial activity.

With respect to turbidity, the raw effluent presented a value of 117.67 ± 1.15 NTU, exceeding the limit of 100 NTU established by CONAMA Resolution 357/2005 for Class II waters. After physicochemical treatment, turbidity was reduced to 36.44 ± 12.29 NTU, corresponding to a removal efficiency of 69.03%, and remaining fully compliant with the legislation.

Lower removal efficiencies were observed for parameters associated with dissolved salts. Electrical conductivity decreased from 7996.67 ± 96.09 mS/cm in the raw effluent to 6473.33 ± 45.09 mS/cm after treatment, corresponding to a reduction of 19.05%. Similarly, total dissolved solids (TDS) were reduced from 6557.27 ± 78.79 mg/L to 5308.13 ± 36.98 mg/L, also with a removal efficiency of 19.05%. Despite this reduction, TDS values remained well above the maximum limit of 500 mg/L established by CONAMA Resolution 357/2005 for Class I, II, and III waters. Regarding conductivity and total dissolved solids, the European Union establishes a limit of 250–1000 μS/cm for conductivity, and the United States a limit of 250–2500 μg/L for TDS. High conductivity and TDS values indicate the persistence of a high ionic load in the treated effluent, suggesting the need for complementary treatment steps. Typically, natural waters present conductivity values between 10 and 100 μS/cm, whereas domestic sewage and industrial effluents may reach values up to 1000 μS/cm.

Electrical conductivity is closely associated with the presence of dissolved cations and anions, mainly calcium, magnesium, sodium, bicarbonates, chlorides, and sulfates. Among these, sodium showed particularly high concentrations, decreasing from 2045.00 ± 0.01 mg/L in the raw effluent to 1819.33 ± 31.94 mg/L after treatment, representing a modest removal efficiency of 11.04%. Potassium concentrations were also reduced from 7.20 ± 0.01 mg/L to 5.27 ± 1.03 mg/L, corresponding to a removal efficiency of 26.81%. No regulatory limits are established for sodium and potassium in CONAMA Resolution 430/2011 or CONSEMA Resolution 355/2017.

For phosphorus, the raw effluent presented a concentration of 17.39 ± 0.75 mg/L, which was reduced to 4.43 ± 0.77 mg/L after treatment, corresponding to a removal efficiency of 74.51%. According to CONSEMA Resolution 355/2017, for effluent discharge flows below 100 m^3^/day, phosphorus concentrations of up to 4 mg/L or a minimum removal efficiency of 75% are permitted. Thus, although the removal efficiency approached the regulatory requirement, the final phosphorus concentration slightly exceeded the established concentration limit. In contrast, CONAMA Resolution 357/2005 establishes much stricter limits for Class II freshwater, ranging from 0.030 to 0.050 mg/L. For phosphorus, the United States and the European Union set limits of 0.1–1.0 mg/L and 1.0–2.0 mg/L, respectively.

Regarding total iron, concentrations decreased from values above 6.00 mg/L in the raw effluent to 2.71 ± 1.59 mg/L after treatment. Although the limited analytical range prevented accurate calculation of removal efficiency, the post-treatment iron concentration remained well below the maximum limit of 10 mg/L established by CONSEMA Resolution 355/2017. For iron, United States legislation provides a disposal limit of up to 1 mg/L. For chlorides, concentrations showed only a slight reduction, from 1514.67 ± 20.50 mg/L to 1420.00 ± 35.50 mg/L (6.25% removal), remaining well above the limit of 250 mg/L established by CONAMA Resolution 357/2005 for Class II waters. For chlorides, European Union legislation also sets a limit of 250 mg/L, while the United States sets it at 230 mg/L.

The removal of phosphorus and iron during the physicochemical treatment step also indicates potential for resource recovery. Phosphorus is considered a critical nutrient in the circular water economy due to its limited global reserves and high demand in agriculture.

Finally, settleable solids remained at 0.01 ± 0.01 mL/L both before and after treatment, complying with the maximum value of 1 mL/L established by CONAMA Resolution 430/2011. However, due to the detection limit of the analytical method employed, it was not possible to calculate the removal efficiency for this parameter.

### Treatment Proposal and Future Perspective

Based on the results of the effluent characterization and the efficiency observed in the Jar-Test assays, a proposal for industrial effluent treatment is presented below. Prior to treatment, pH adjustment is required using a 25% sulfuric acid solution in order to establish optimal conditions for coagulation and flocculation. The primary physicochemical treatment consists of coagulation, flocculation, and sedimentation. Coagulation corresponds to the rapid mixing stage (100 rpm for 2 min and 30 s), during which aluminum sulfate (50% solution) is added to destabilize colloidal particles and promote particle aggregation. Flocculation is carried out under slow mixing conditions (50 rpm for 25 min), allowing suspended particles to agglomerate and form flocs that subsequently settle by gravity. This treatment stage is mainly responsible for the reduction of turbidity (suspended particles) and color (dissolved particles), as well as significant decreases in total alkalinity, hardness, COD, and nutrients such as phosphorus. These unit operations may be implemented in hydraulic or mechanically mixed systems, with the final configuration selected based on a cost–benefit analysis. Figure 5 presents a flowchart with the proposed treatment for the effluent in question and reach the fit-for-purpose water reuse.

**Figure 5:**
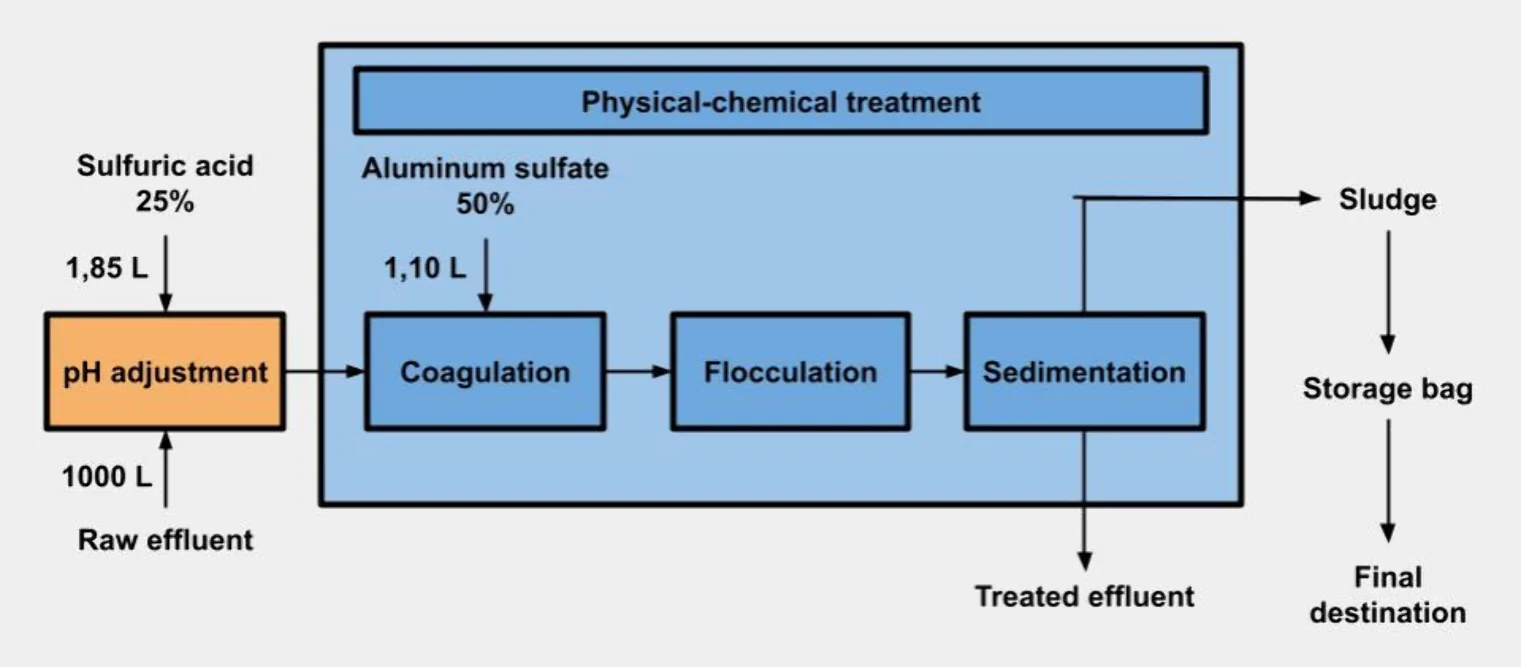
Flowchart of the proposed treatment for the effluent in question.

Despite its effectiveness, the primary physicochemical treatment alone is not sufficient to significantly reduce conductivity and total dissolved solids, which, in this study, are strongly associated with elevated concentrations of sodium and chlorides (originating from formulations that contain compounds such as sodium hydroxide, sodium hypochlorite, and ferric chloride). Therefore, as a future perspective, an additional polishing step, such as ion exchange or reverse osmosis, may be considered to further reduce these specific ions. Because these advanced polishing technologies were not experimentally evaluated at the bench scale in the present study, more detailed future investigations are required to determine which method is most efficient and economically viable. Ultimately, the proposed primary treatment system can be scaled according to the required processing capacity, enabling the treated effluent to be reused in industrial operations requiring nonpotable water, thus reducing freshwater abstraction. Such integration of treatment and resource recovery strategies represents an important step toward circular water management in industrial facilities.

### Preliminary Technoeconomic Analysis (TEA) for Full-Scale Implementation

An estimate of the chemical costs required for full-scale effluent treatment was conducted based on an effluent volume of 1000 L. The required amounts of aluminum sulfate and sulfuric acid were extrapolated from the doses determined in the Jar-Test assays, and their masses were estimated using density relationships.

The mass of aluminum sulfate required for treating 1000 L of effluent was calculated to be approximately 1.43 kg, corresponding to a cost of 1.83 in Brazilian reais (∼ 0.35 in US dollars) based on a unit price of 1.28 per kg in Brazilian reais. The sulfuric acid consumption was estimated using the prepared 25% solution required for pH adjustment, resulting in an equivalent of approximately 0.86 kg of commercial 98% sulfuric acid. At a unit cost of 4.35 in Brazilian reais per kg, this corresponds to an expenditure of 3.76 in Brazilian reais (∼ 0.71 in US dollars).

Therefore, the total estimated chemical cost to treat 1000 L of effluent using the proposed physicochemical treatment is approximately 5.59 in Brazilian reais (∼ 1.06 in US dollars). On the basis of an average effluent generation rate of 2000 L per day at the facility, this chemical cost scales to approximately 11.18 in Brazilian reais (∼ 2.12 in US dollars) per operating day. Assuming 252 operating days per year, the annual cost associated with chemical inputs for the physicochemical treatment is estimated at 2817.36 in Brazilian reais (∼ 533 in US dollars).

By comparing the chemical treatment cost (∼ 1.06 in US dollars per 1000 L) with the current industrial tariff for fresh potable water, the economic viability of this circular approach becomes evident, not only reducing water intake but also providing financial savings.

This cost estimate does not include capital expenditures (CAPEX) for equipment, labor, energy, maintenance, or disposal of treatment residues, which should be evaluated in a comprehensive economic analysis. Similarly, sensitivity to fluctuations in reagent prices and foreign exchange rates should be considered when projecting long-term operational costs.

## Conclusions

Water is widely used in industrial processes and frequently acquires physicochemical characteristics that prevent its direct discharge into receiving water bodies. Consequently, efficient wastewater treatment is essential to ensure regulatory compliance and minimize environmental impacts. In this study, a treatability assessment was conducted to characterize raw and treated effluents and to evaluate the efficiency of physicochemical treatment at the laboratory scale. Additionally, mapping the production process proved to be a crucial step, as it enabled the identification of effluent-generating stages and their respective contributions to the overall effluent composition.

The results demonstrated that coagulation–flocculation using aluminum sulfate significantly improved effluent quality, with substantial reductions observed in several key physicochemical parameters. The optimal operating conditions were identified as a dosage of 55 mg/L of 50% aluminum sulfate at an initial pH between 8 and 9, considering both regulatory requirements and cost–effectiveness.

Overall, the findings highlight the importance of specific treatability studies in defining optimal treatment configurations for industrial effluents. By establishing these operational parameters, the proposed treatment approach supports direct compliance with environmental discharge regulations and provides a reliable technical baseline for the industry’s wastewater management. Despite the promising results, this study presents limitations inherent to laboratory-scale experiments. Future investigations should evaluate the optimized treatment system at a pilot or full industrial scale, including assessments of continuous flow performance, operational robustness, and long-term economic feasibility.

## Supplementary Material



## Notes

Calibration curves for sodium and potassium determination by flame spectrophotometry; typical coagulation diagram of aluminum sulfate; ANOVA statistical analysis tables for pH, turbidity, and total alkalinity from jar-test experiments (PDF)
