# Supplementary material for: Optimization of Coagulation-Flocculation with Aluminum Sulfate for the Treatment of Chemical Industry Effluent
Source: ACS Omega. 2026 Sep 28;11(40):61662–73. doi: 10.1021/acsomega.6c06781 (PMC13670290; doi:10.1021/acsomega.6c06781)
Supplement: Supplementary file 1 [file ao6c06781_si_001.pdf]

SUPPORTING INFORMATION

Optimization of coagulation-flocculation with aluminum sulfate for the treatment of chemical industry effluent

Sabrina G. Juchem, Suyanne A. L. Bachmann, Fellipe F. S. Farias*

Federal Institute of Education, Science and Technology of Rio Grande do Sul (IFRS), Feliz Campus, 95770-000, RS, Brazil.

**E-mail: fellipefreire@hotmail.com*

SUMMARY

Appendix A – Calibration curves obtained in the analysis of sodium and potassium by flame spectrophotometry. 2

Annex A – Typical Coagulation Diagram of Aluminum Sulfate. 6

Appendix B – Results of anovas for the *jar-test* tests. 7

Appendix A – Calibration curves obtained in the analysis of sodium and potassium by flame spectrophotometry.

Figure S1 – Calibration curve for sodium in treated effluent sample **1**.

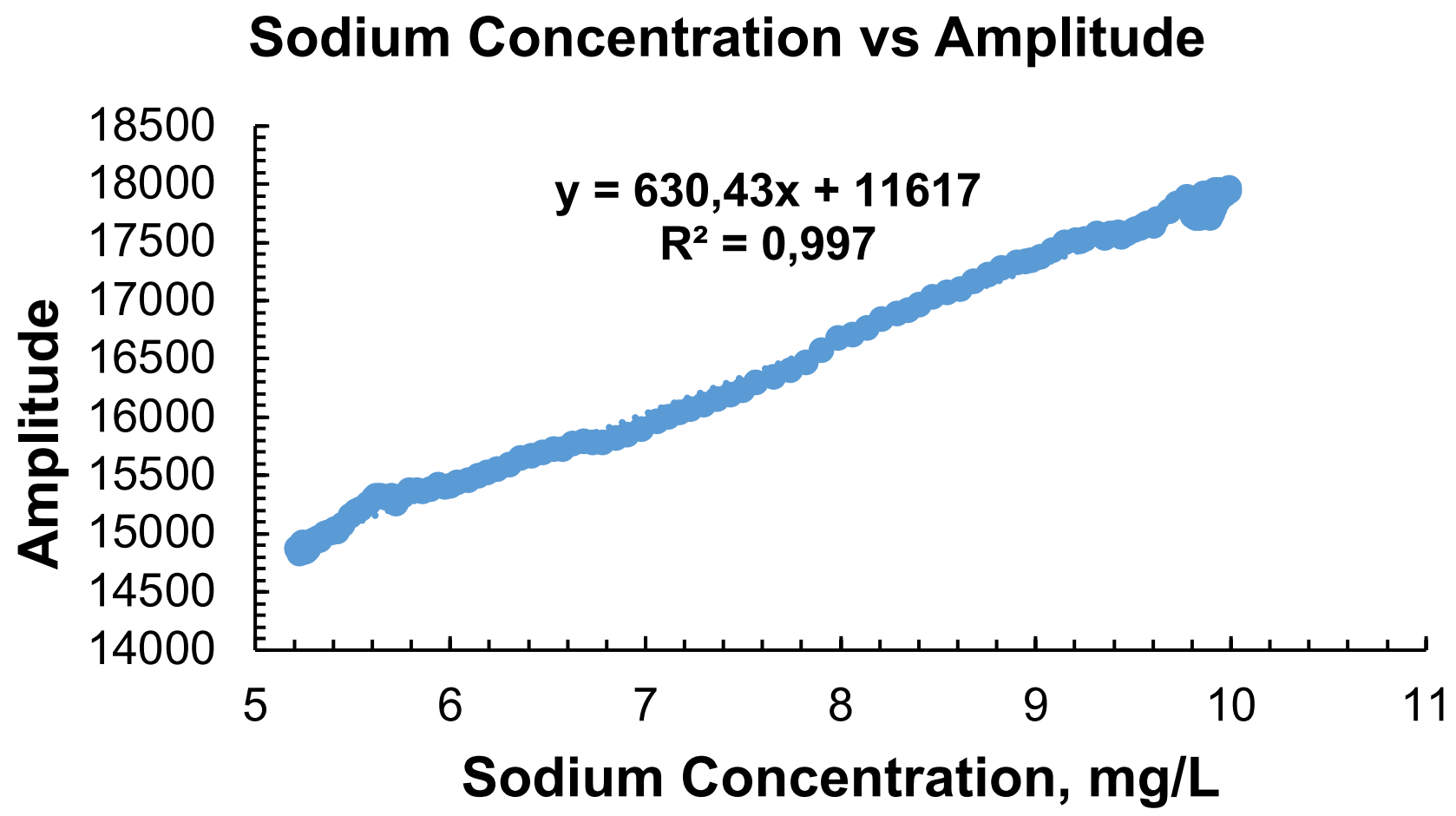

Source: Author (2025).

Figure S2 – Calibration curve for sodium in treated effluent sample **2**.

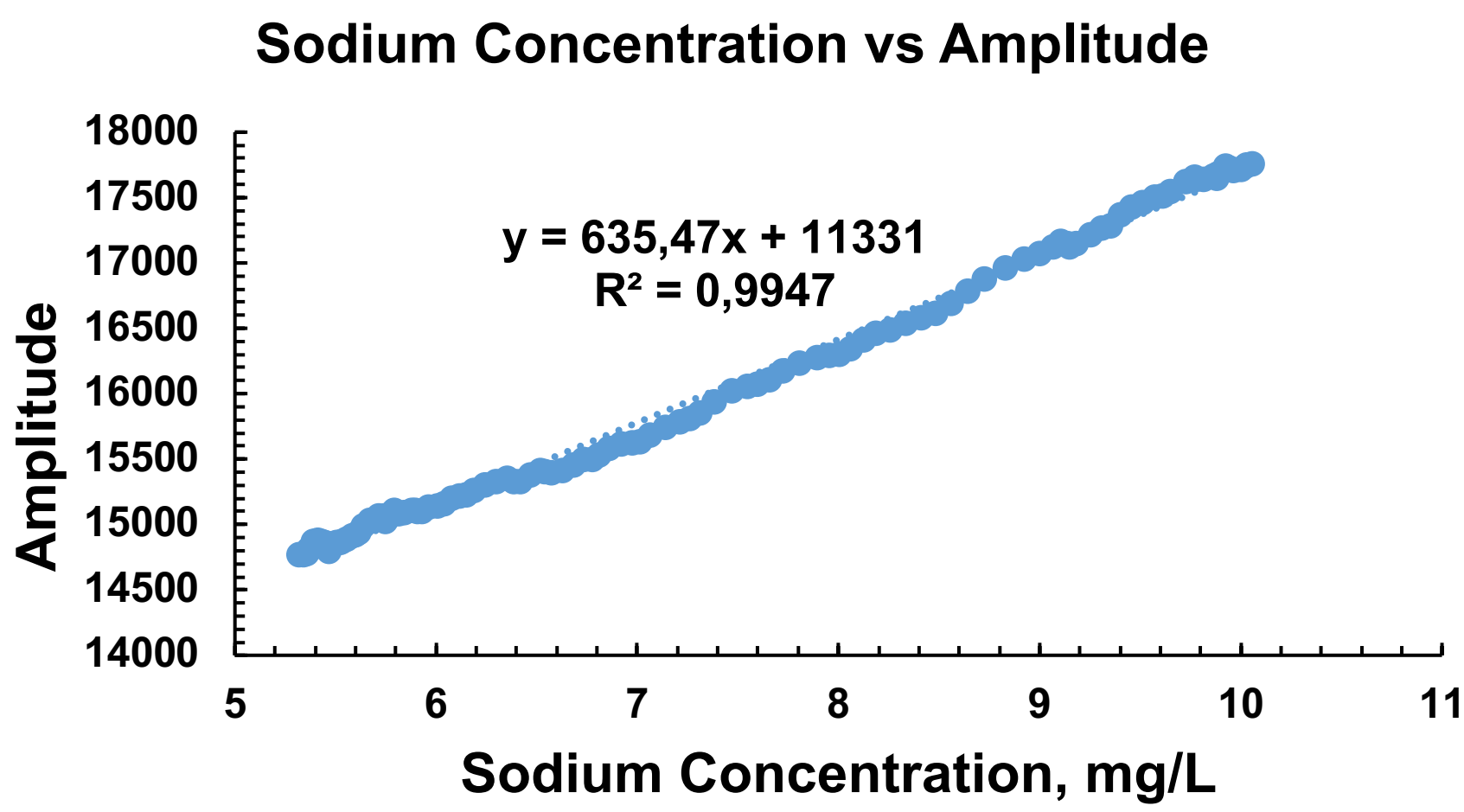

Source: Author (2025).

Figure S3 – Calibration curve for sodium in treated effluent sample **3**.

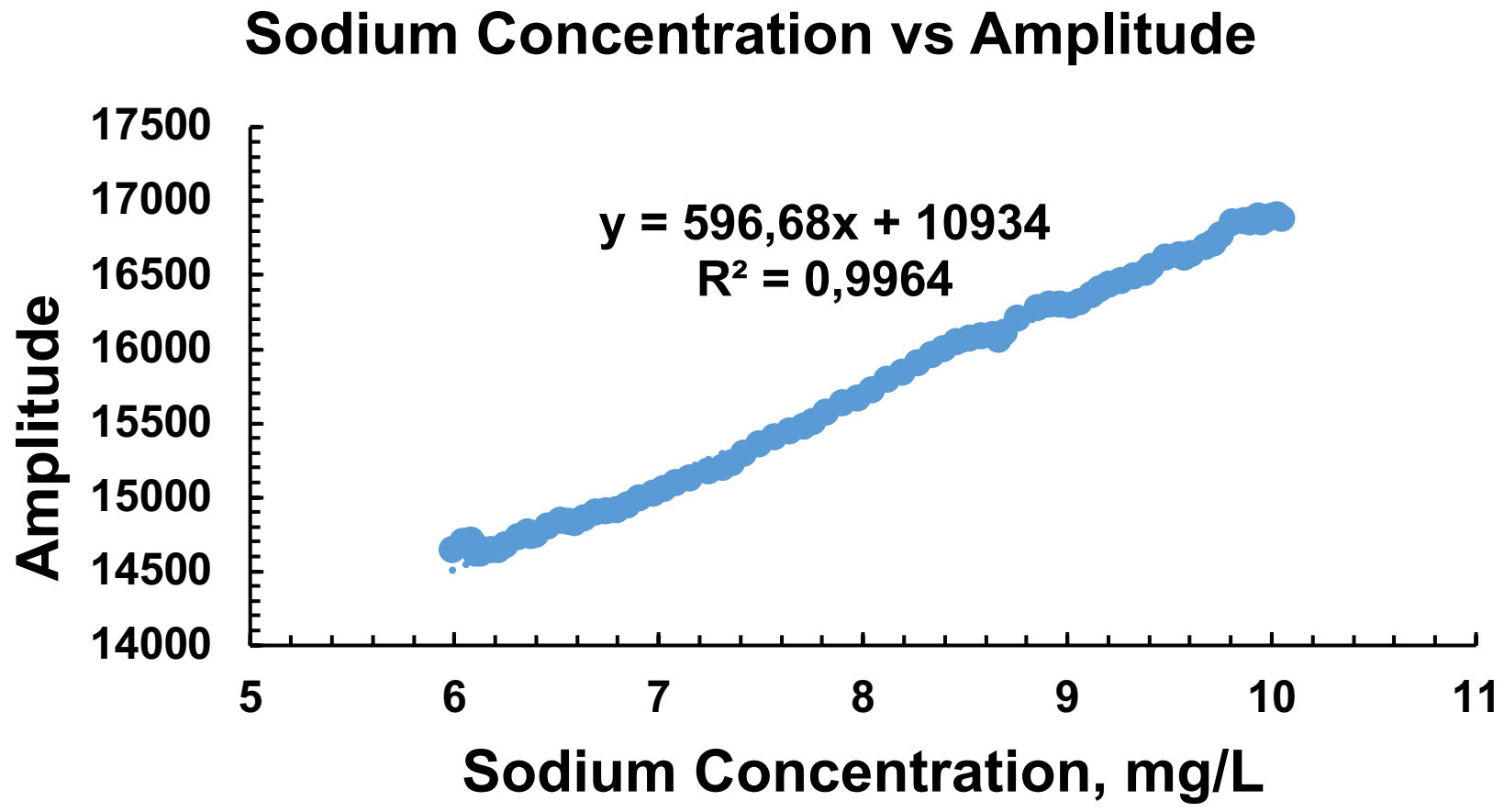

Source: Author (2025).

Figure S4 – Calibration curve for potassium in treated effluent sample **1**.

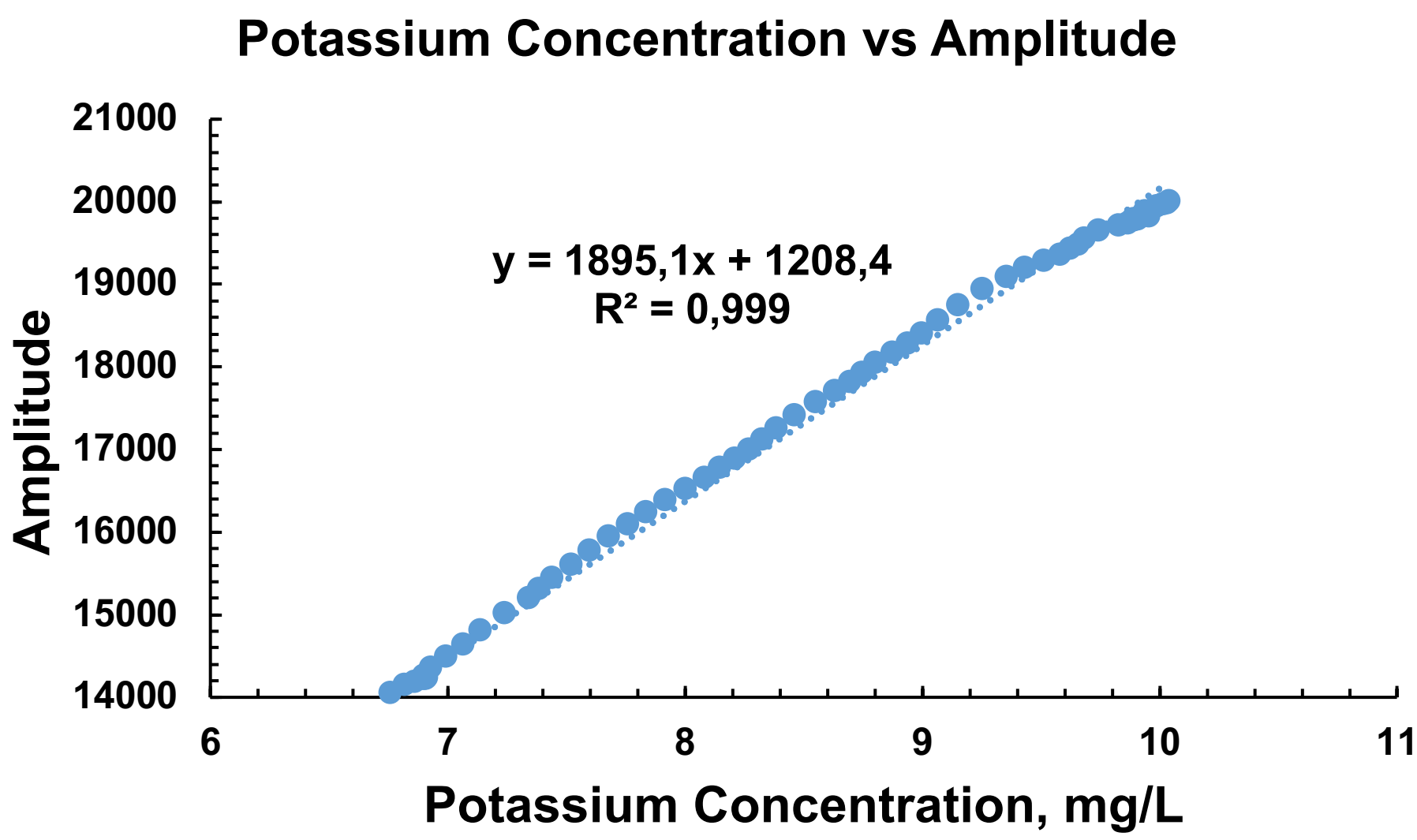

Source: Author (2025).

Figure S5 – Calibration curve for potassium in treated effluent sample **2.**

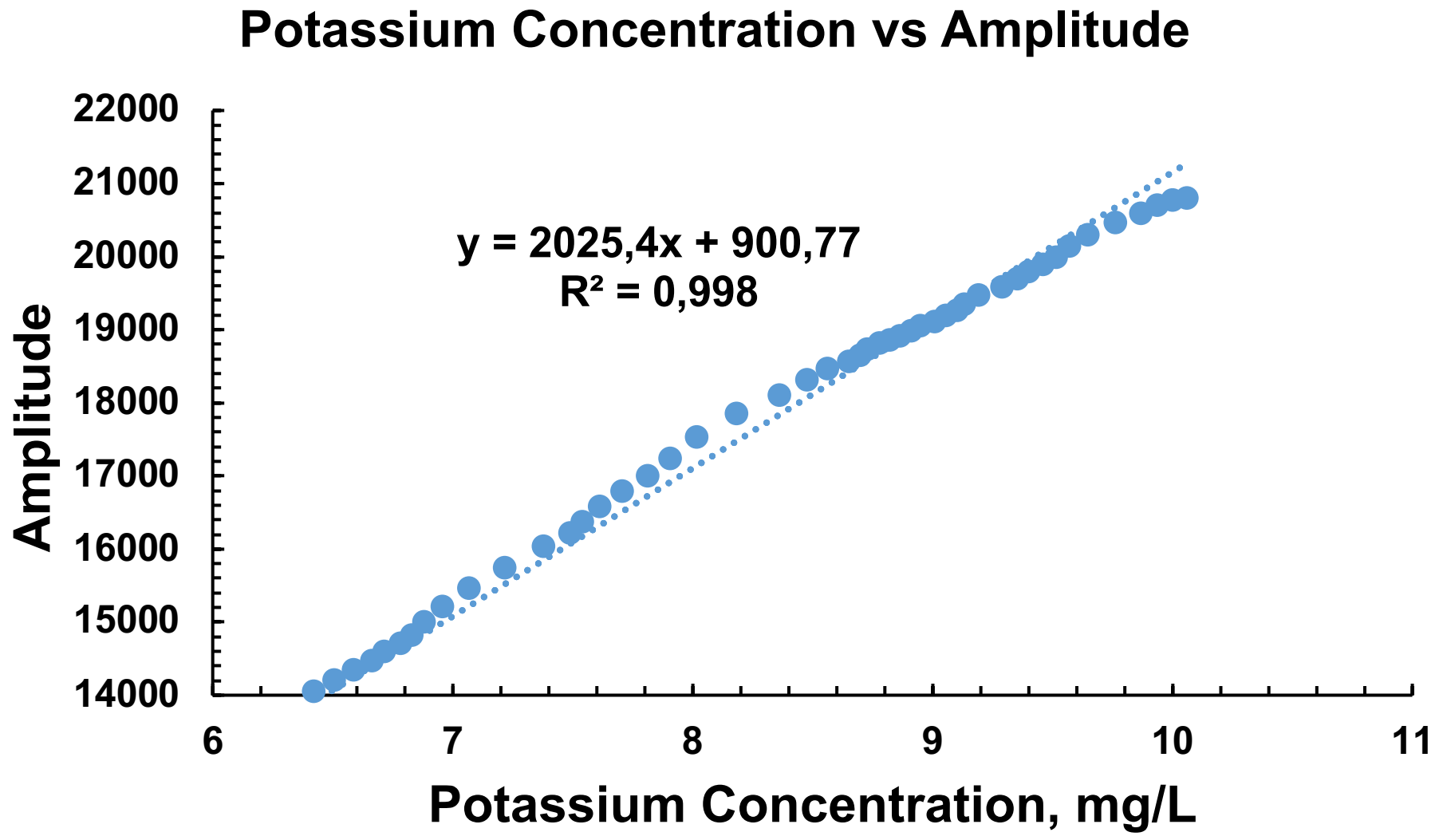

Source: Author (2025).

Figure S6 – Calibration curve for potassium in treated effluent sample **3.**

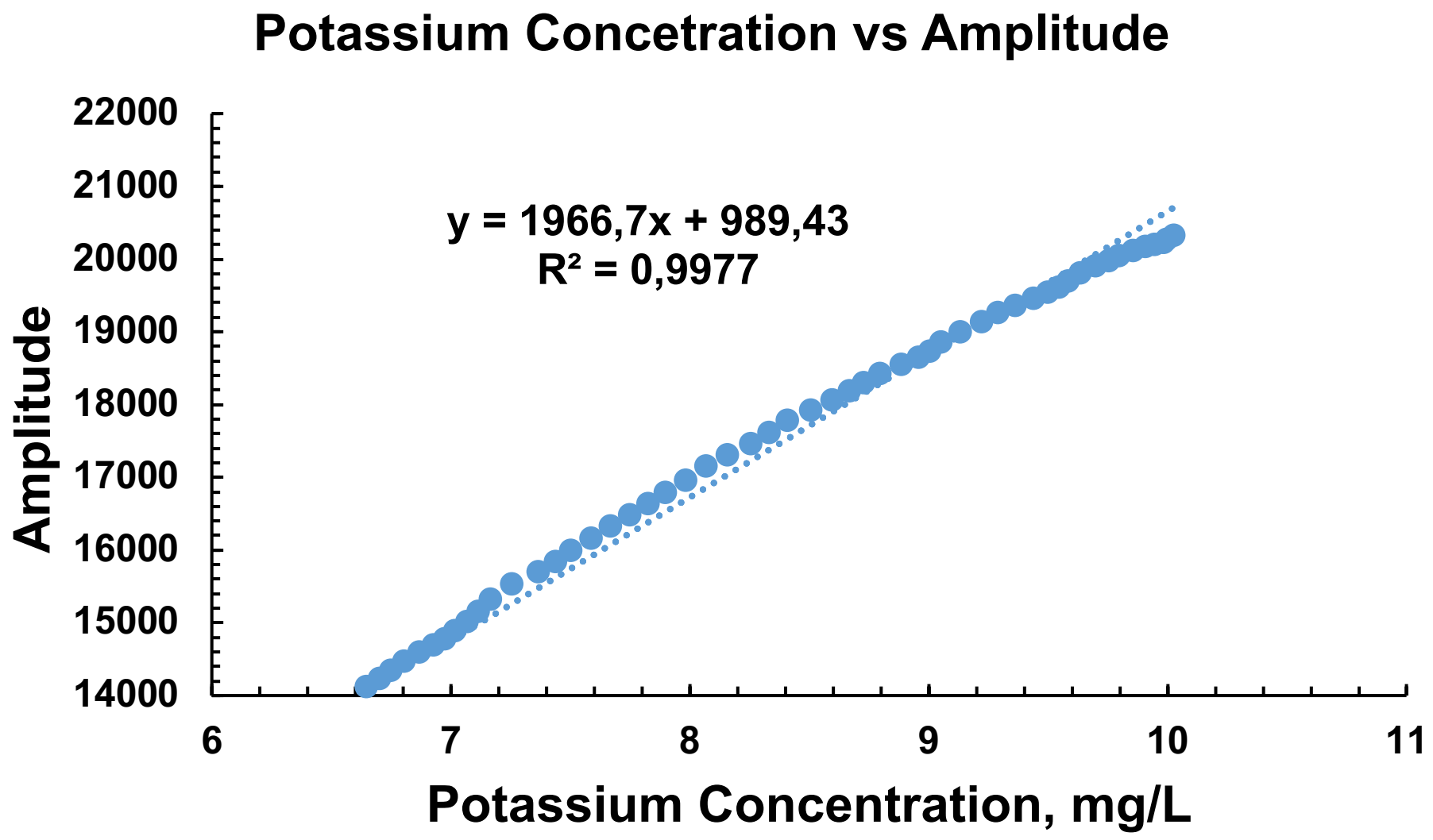

Source: Author (2025).

Figure S7 – Calibration curve for sodium in the raw effluent sample.

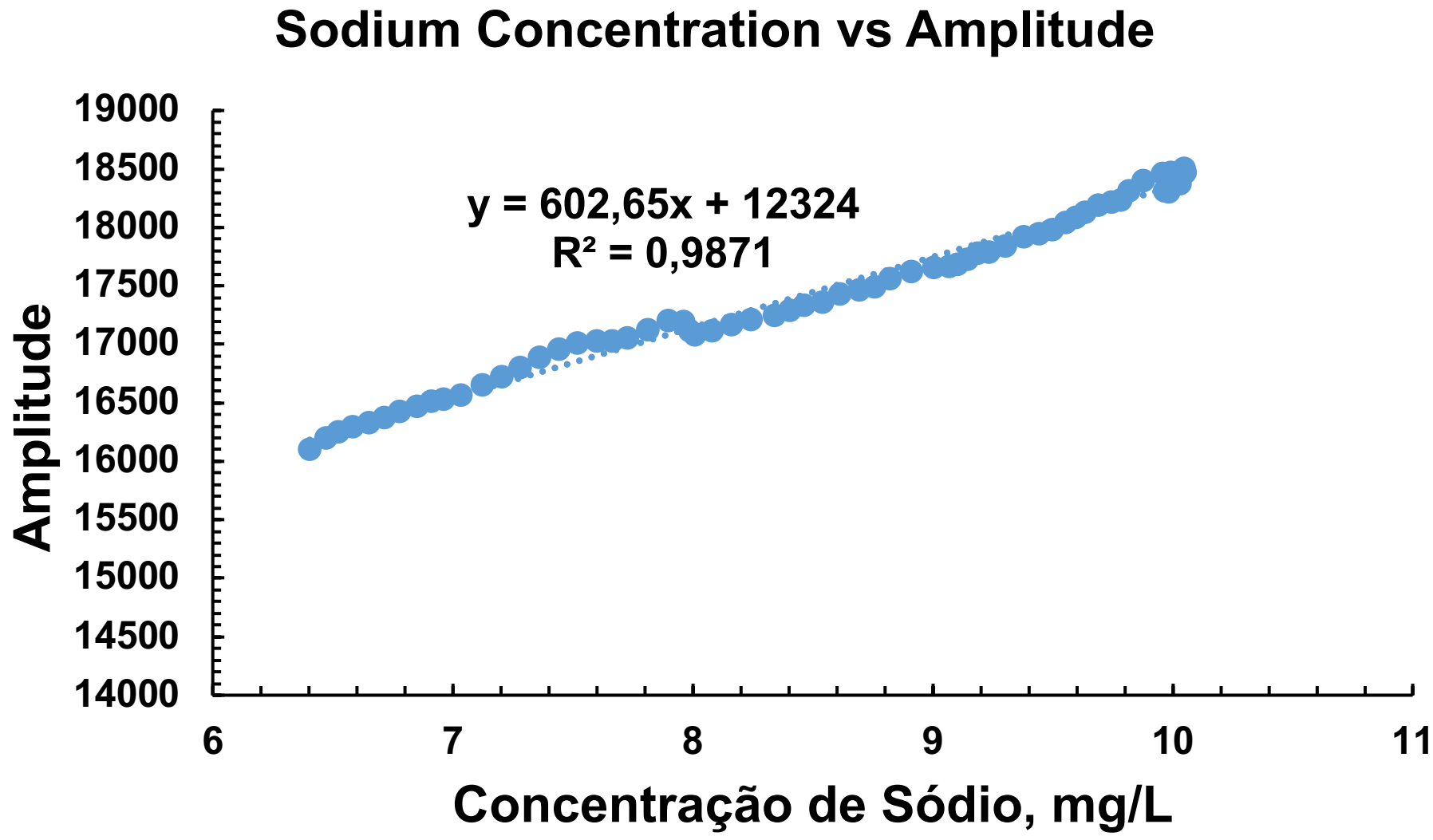

Source: Author (2025).

Figure S8 – Calibration curve for potassium in the raw effluent sample.

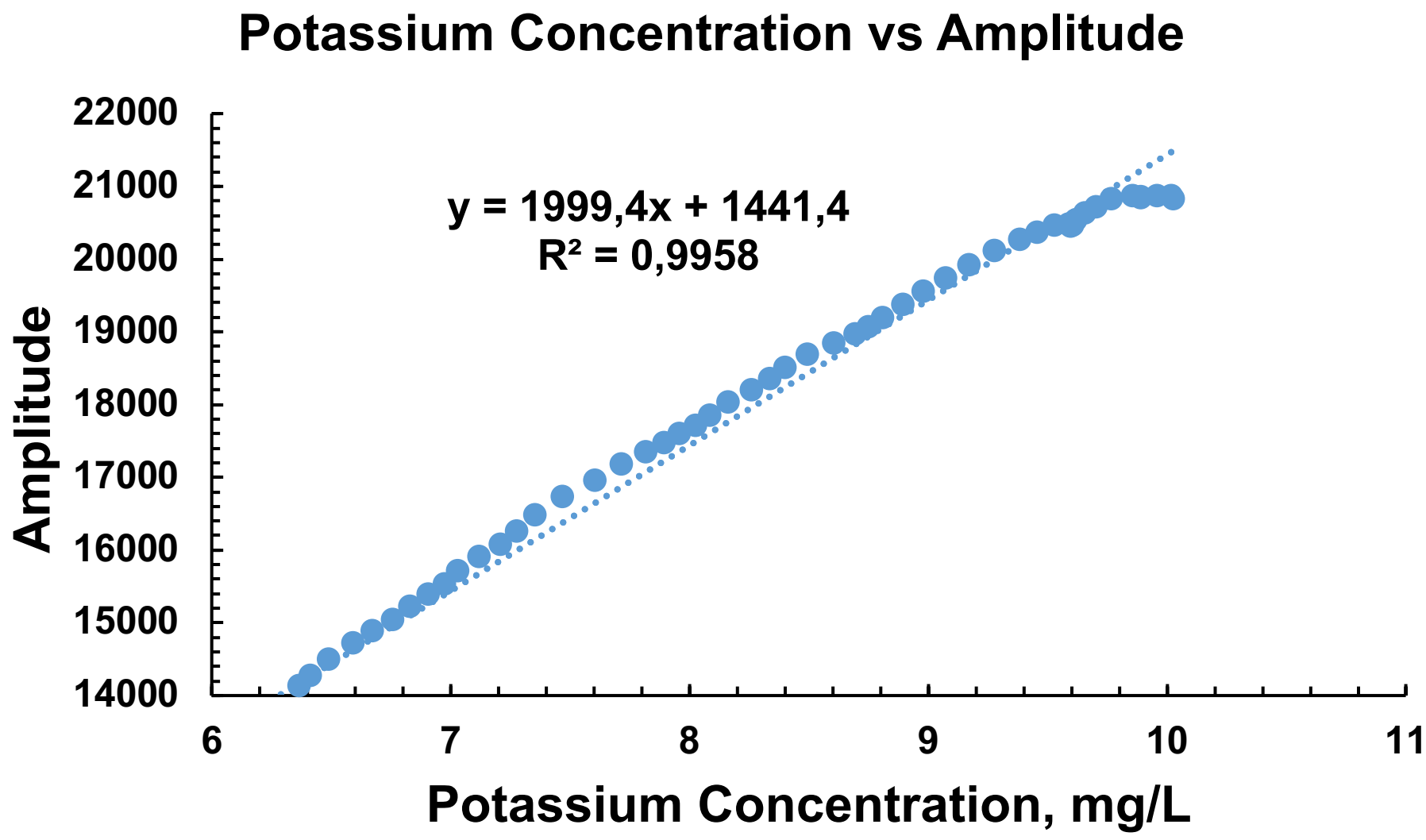

Source: Author (2025).

Annex A – Typical Coagulation Diagram of Aluminum Sulfate.

Figure S9. Typical coagulation diagram of aluminum sulfate.

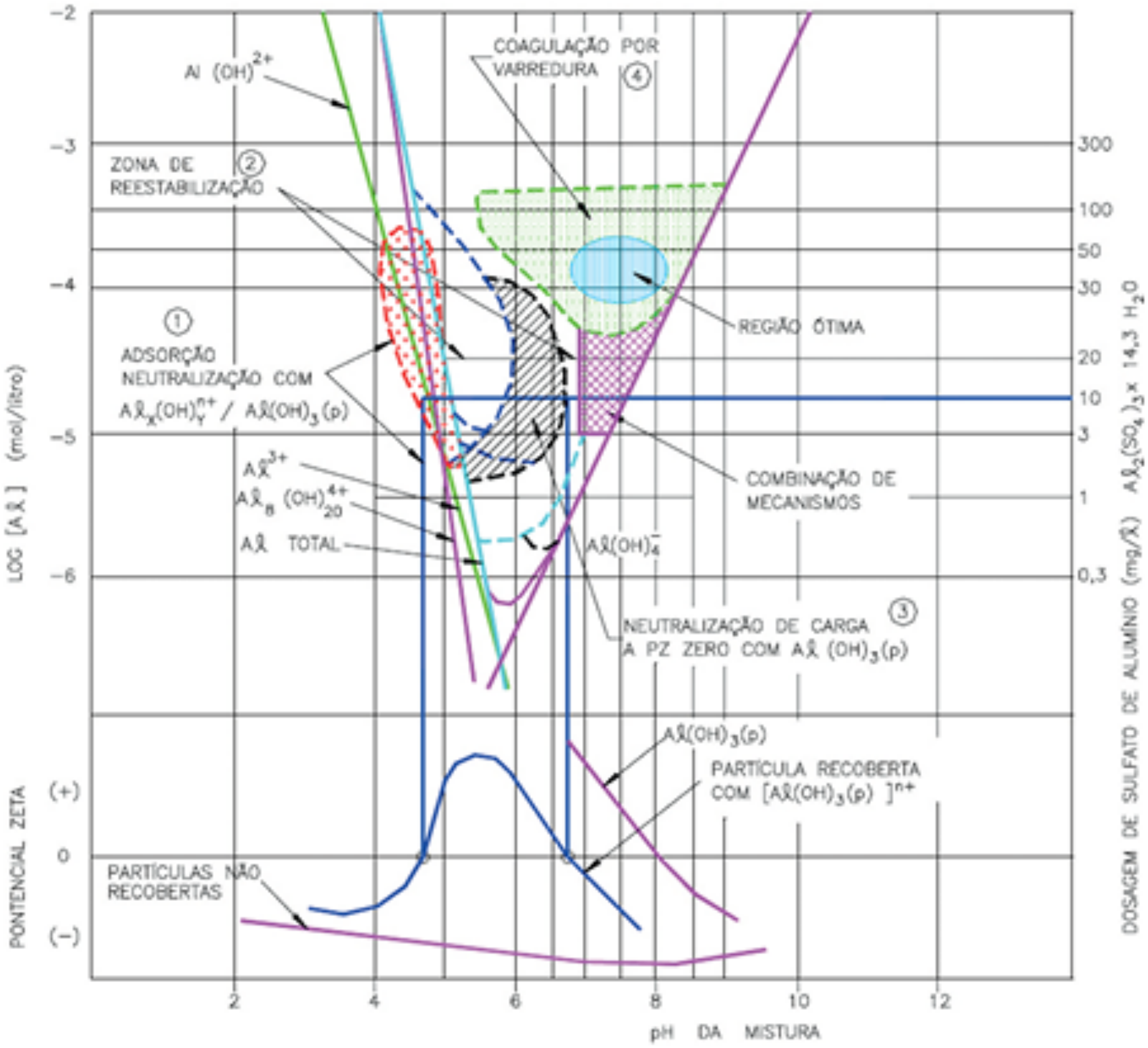

Source: Amirtharajah e Mills, (1982) *apud* Di Bernardo, (2005).

Appendix B – Results of ANOVA for the jar-test tests.

Table S1 – ANOVA comparing the pH of jars 5 and 6.

Entry	Dosage, mg/L	final pH		
5	45	5.98	5.97	5.80
6	50	5.61	5.59	5.58

ANOVA: single factor.

SUMMARY

Group	*Count*	*Sum*	*Average*	*Variance*
45	3	17.75	5.9166667	0.010233333
50	3	16.78	5.5933333	0.000233333

ANOVA

Source of variation	*SQ*	*gl*	*MQ*	*F*	*p-value*	*Critical F*
Between groups	0.156816667	1	0.1568167	29.96496815	0.005419883	7,708647422
Within the groups	0.020933333	4	0.0052333			
Total	0.17775	5				

Source: Author (2025).

Table S2 – ANOVA comparing the turbidity of jars 5 and 6.

Entry	Dosage, mg/L	Final turbidity (UNT)		
5	45	5.94	5.25	6.00
6	50	3.52	4.35	5.91

ANOVA: single factor.

SUMMARY

Group	*Count*	*Sum*	*Average*	*Variance*
45	3	17,19	5.73	0.1737
50	3	13.78	4.593333333	1.472433333

ANOVA

Source of variation	*SQ*	*gl*	*MQ*	*F*	*p-value*	*Critical F*
Between groups	1.93801667	1	1.938016667	2.35462903	0.199699	7,708647
Within the groups	3.29226667	4	0.823066667			
Total	5.23028333	5				

Source: Author (2025).

Table S3 – ANOVA comparing the total alkalinity of jars 5 and 6.

**Entry**	**Dosage, mg/L**	**Final Total Alkalinity (ppm CaCO3)**		
5	45	130.00	125.00	135.00
6	50	85.00	90.00	95.00

ANOVA: single factor.

SUMMARY

Group	*Count*	*Sum*	*Average*	*Variance*
45	3	390	130	25
50	3	270	90	25

ANOVA

Source of variation	*SQ*	*gl*	*MQ*	*F*	*p-value*	*Critical F*
Between groups	2400	1	2400	96	0.000608	7,708647
Within the groups	100	4	25			
Total	2500	5				

Source: Author (2025).

Table S4 – ANOVA comparing the pH of jars 9 and 10.

Entry	Dosage, mg/L	final pH		
9	55	7.07	7.04	7.03
10	60	7.37	7.37	7.21

ANOVA: single factor.

SUMMARY

Group	*Count*	*Sum*	*Average*	*Variance*
55	3	21.14	7.046667	0.000433
60	3	21.95	7.316667	0.008533

ANOVA

Source of variation	*SQ*	*gl*	*MQ*	*F*	*p-value*	*Critical F*
Between groups	0.10935	1	0.10935	24.39033	0.007824	7,7086474
Within the groups	0.017933333	4	0.004483			
Total	0.127283333	5				

Source: Author (2025).

Table S5 – ANOVA comparing the turbidity of jars 9 and 10.

**Entry**	**Dosage, mg/L**	**Final turbidity (UNT)**		
9	55	22.70	27.50	29.40
10	60	48.10	48.00	50.20

ANOVA: single factor.

SUMMARY

Group	*Count*	*Sum*	*Average*	*Variance*
55	3	79.6	26.53333333	11.92333
60	3	146.3	48.76666667	1.543333

ANOVA

Source of variation	*SQ*	*gl*	*MQ*	*F*	*p-value*	*Critical F*
Between groups	741.4817	1	741,4816667	110,121	0.000466	7,708647
Within the groups	26.93333	4	6.733333333			
Total	768,415	5				

Source: Author (2025).

Table S6 – ANOVA comparing the total alkalinity of jars 9 and 10.

Entry	Dosage, mg/L	Final Total Alkalinity (ppm $CaCO_3$)		
9	55	260.00	275.00	270.00
10	60	295.00	305.00	285.00

ANOVA: single factor.

SUMMARY

Group	*Count*	*Sum*	*Average*	*Variance*
55	3	805	268.3333	58.33333
60	3	885	295	100

ANOVA

Source of variation	*SQ*	*gl*	*MQ*	*F*	*p-value*	*Critical F*
Between groups	1066,667	1	1066,667	13.47368	0.021379	7,708647
Within the groups	316.6667	4	79.16667			
Total	1383,333	5				

Source: Author (2025).

Table S7 – ANOVA comparing the pH of jars 5, 6, 9 and 10.

Entry	Dosage, mg/L	final pH		
5	45	5.98	5.97	5.80
6	50	5.61	5.59	5.58
9	55	7.07	7.04	7.03
10	60	7.37	7.37	7.21

ANOVA: single factor.

SUMMARY

Group	*Count*	*Sum*	*Average*	*Variance*
45	3	17.75	5.91666667	0.010233333
50	3	16.78	5.59333333	0.000233333
55	3	21.14	7.04666667	0.000433333
60	3	21.95	7.31666667	0.008533333

ANOVA

Source of variation	*SQ*	*gl*	*MQ*	*F*	*p-value*	*Critical F*
Between groups	6.3723	3	2.1241	437.2075472	3,3159E-09	4.06618055
Within the groups	0.0388667	8	0.00485833			
Total	6.4111667	11				

Source: Author (2025).

Table S8 – ANOVA comparing the turbidity of jars 5, 6, 9 and 10.

Entry	Dosage, mg/L	Final turbidity (UNT)		
5	45	5.94	5.25	6.00
6	50	3.52	4.35	5.91
9	55	22.70	27.50	29.40
10	60	48.10	48.00	50.20

ANOVA: single factor.

SUMMARY

Group	*Count*	*Sum*	*Average*	*Variance*
45	3	17,19	5.73	0.1737
50	3	13.78	4.593333	1.47243333
55	3	79.6	26.53333	11.9233333
60	3	146.3	48.76667	1.54333333

ANOVA

Source of variation	*SQ*	*gl*	*MQ*	*F*	*p-value*	*Critical F*
Between groups	3909,895	3	1303,298	344.95219	8.5E-09	4.066181
Within the groups	30.2256	8	3.7782			
Total	3940,121	11				

Source: Author (2025).

Table S9 – ANOVA comparing the total alkalinity of jars 5, 6, 9 and 10.

Entry	Dosage, mg/L	Final Total Alkalinity (ppm $CaCO_3$)		
5	45	130.00	125.00	135.00
6	50	85.00	90.00	95.00
9	55	260.00	275.00	270.00
10	60	295.00	305.00	285.00

ANOVA: single factor.

SUMMARY

Group	*Count*	*Sum*	*Average*	*Variance*
45	3	390	130	25
50	3	270	90	25
55	3	805	268.3333	58.33333
60	3	885	295	100

ANOVA

Source of variation	*SQ*	*gl*	*MQ*	*F*	*p-value*	*Critical F*
Between groups	91875	3	30625	588	1.02E-09	4.066181
Within the groups	416.6667	8	52.08333			
Total	92291.67	11				

Source: Author (2025).

Table S10 – ANOVA comparing the pH of jars 6 and 9.

Entry	Dosage, mg/L	final pH		
6	50	5.61	5.59	5.58
9	55	7.07	7.04	7.03

ANOVA: single factor.

SUMMARY

Group	*Count*	*Sum*	*Average*	*Variance*
50	3	16.78	5.593333	0.000233
55	3	21.14	7.046667	0.000433

ANOVA

Source of variation	*SQ*	*gl*	*MQ*	*F*	*p-value*	*Critical F*
Between groups	3.168267	1	3.168267	9504.8	6.64E-08	7,708647
Within the groups	0.001333	4	0.000333			
Total	3.1696	5				

Source: Author (2025).

Table S11 – ANOVA comparing the turbidity of jars 6 and 9.

Entry	Dosage, mg/L	Final turbidity (UNT)		
6	50	3.52	4.35	5.91
9	55	22.7	27.5	29.4

ANOVA: single factor.

SUMMARY

Group	*Count*	*Sum*	*Average*	*Variance*
50	3	13.78	4.593333333	1.47243333
55	3	79.6	26.53333333	11.9233333

ANOVA

Source of variation	*SQ*	*gl*	*MQ*	*F*	*p-value*	*Critical F*
Between groups	722.0454	1	722.0454	107,802027	0.000486	7,708647
Within the groups	26.79153	4	6.697883333			
Total	748.8369	5				

Source: Author (2025).

Table S12 – ANOVA comparing the alkalinity of jars 6 and 9.

Entry	Dosage, mg/L	Final Total Alkalinity (ppm $CaCO_3$)		
6	50	85	90	95
9	55	260	275	270

ANOVA: single factor.

SUMMARY

Group	*Count*	*Sum*	*Average*	*Variance*
50	3	270	90	25
55	3	805	268.3333	58.33333

ANOVA

Source of variation	*SQ*	*gl*	*MQ*	*F*	*p-value*	*Critical F*
Between groups	47704.17	1	47704.17	1144.9	4.55E-06	7,708647
Within the groups	166.6667	4	41.66667			
Total	47870.83	5				

Source: Author (2025).
